# Bioengineering DNA-based hydrogels for regenerative medicine: A review of programmable design, chemical synthesis and therapeutic potential

**DOI:** 10.1016/j.mtbio.2026.103448

**Published:** 2026-07-12

**Authors:** Shihui Xu, Eon-Bee Lee, Yiming Shen, Yongtao Wang, Xue Zhang

**Affiliations:** a School of Medicine, Shanghai University, Shanghai, 200444, China; b Department of Aquatic Life Medicine, Pukyong National University, Busan, 48513, South Korea; c Department of Pharmacy, China Pharmaceutical University, Nanjing, 210009, China

**Keywords:** DNA hydrogels, Bioactive materials, Functionality, Regenerative medicine, Therapeutic potential

## Abstract

DNA hydrogels have emerged as a transformative class of intelligent biomaterials for regenerative medicine, leveraging the unique programmability, precise molecular recognition, and excellent biocompatibility. Although substantial progress has been made in the development of DNA hydrogels for biomedical applications, a comprehensive understanding about how construction strategies govern physicochemical properties, biological functions, and regenerative medicine remains fragmented. This review systematically examines the molecular design principles, construction strategies, and advanced functionalities of DNA hydrogels, with a focus on their translation into tissue engineering and clinical applications. Physicochemical and biological properties are discussed to underpin their performance. The diverse biomedical applications are further highlighted, particularly for their regenerative potential in repairing bone, cartilage, cardiovascular, skin, and neural tissues. Additionally, they play a crucial role in tumor therapy and the regeneration of tumor-resected tissues. This review may provide a rational framework to guide the development of DNA hydrogels toward precisely regenerative medicine and personalized therapies.

## Highlights

• DNA hydrogels are designed through intrinsic programmability and dynamic sequence control.
• Mechanical adaptability, stimulus responsiveness and biocompatibility of DNA hydrogels are analyzed.
• Construction strategies of pure and hybrid DNA hydrogels are systematically reviewed.
• Regenerative potential of DNA hydrogels is evaluated with therapeutic perspective.

## Graphical abstract

This review provides a systematic framework for DNA hydrogels in regenerative medicine, unifying molecular design, synthesis strategies, and biological functions under the principles of intrinsic programmability and dynamic sequence control. DNA hydrogels are also evaluated preclinical regenerative potential, including bone, cartilage, cardiovascular tissues, skin, neural tissue regeneration and repair, and further play the crucial role in cancer therapy and cancer-resected tissue regeneration. This may guide DNA hydrogels toward precision regenerative medicine and personalized therapy. This figure was created in BioRender.com.

**Figure:**
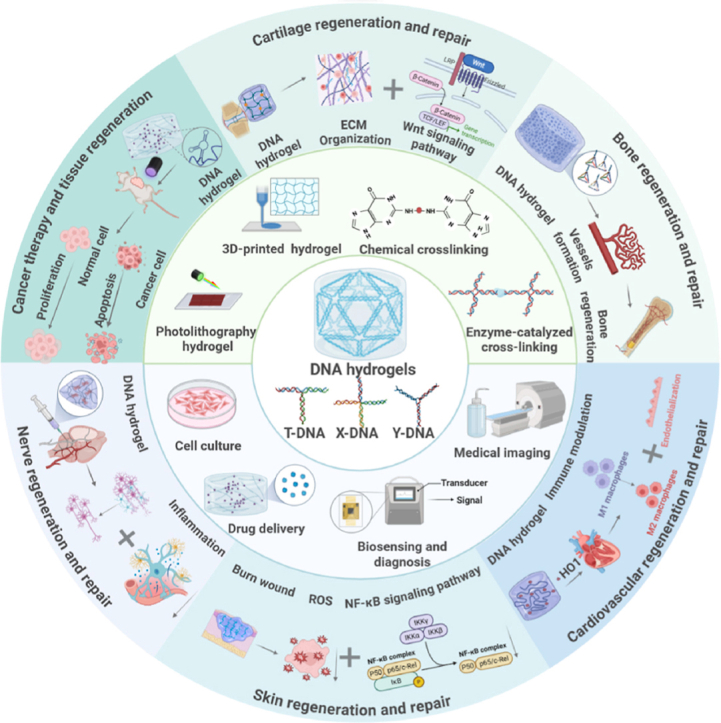

## 1 Introduction

Regenerative medicine has witnessed remarkable advances in tissue engineering, stem cell-based therapies, and bioprinting technologies in recent years [[1], [2], [3], [4]]. In particular, the strategies that leverage artificially engineered extracellular matrix (ECM) models in combination with induced pluripotent stem cells (iPSCs) have demonstrated substantial potential for tissue repair and regeneration, highlighting promising translational and clinical prospects [5,6]. Despite these advances, significant challenges remain in efficient tissue regeneration. A central and unresolved issue lies in the precise recapitulation of the dynamic microenvironment of native ECM, which is essential for achieving spatiotemporally controlled regulation of cell behavior and the regeneration of functional tissues. The native ECM not only provides structural and mechanical support for cells but also orchestrates cell proliferation, differentiation, and migration through a highly complex interplay of biochemical cues and biomechanical properties [7].

**Fig. 1:**
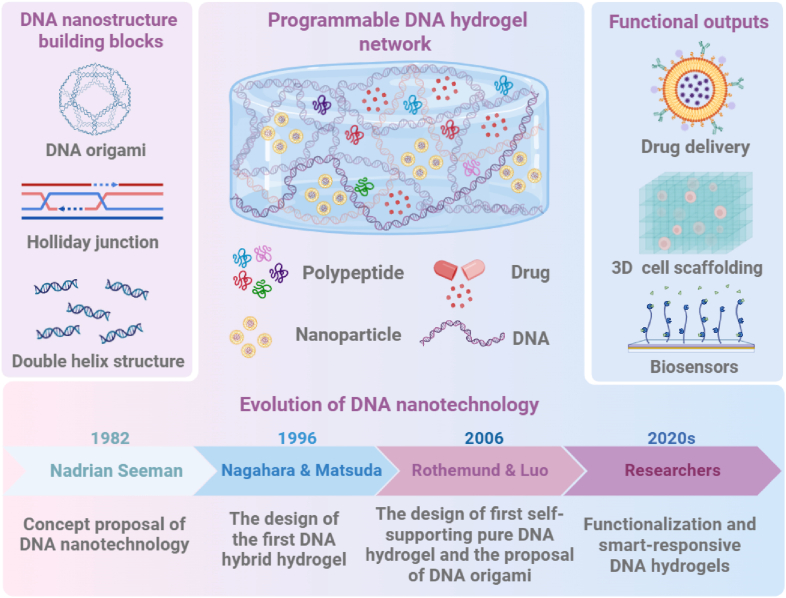
Conceptual illustration to connect molecular building blocks and construction strategies to functional outputs. The introductory timeline is summarized to show the evolution of DNA nanotechnology and hydrogels. This figure was created in BioRender.com.

Since 1982, Nadrian Seeman proposed the concept of DNA nanotechnology and the first DNA hydrogel was designed in 1996 (Fig. 1). DNA hydrogels, as an emerging class of intelligent biomaterials, have attracted increasing attention for their broad potential in regenerative medicine. Genetic material-derived DNA hydrogels leverage the intrinsic precision of Watson-Crick base pairing, exceptional molecular programmability, and favorable biocompatibility, thereby overcoming many inherent limitations of conventional hydrogel systems [[8], [9], [10]]. Importantly, the structural programmability of DNA hydrogels enables precise molecular-level design and regulation [11]. Key physicochemical parameters, including mechanical stiffness, network porosity, and degradation kinetics can be finely regulated based on rational sequence engineering. By modulating pore architecture and degradation behavior, DNA hydrogels provide robust structural and biochemical support for tissue regeneration [12]. In addition, DNA hydrogels can function as immunomodulatory platforms, acting as immune adjuvants that activate the cGAS-STING signaling pathway, thereby enhancing immune responses and contributing to both tissue regeneration and anticancer therapies. As a naturally metabolizable biomaterial with intrinsically low immunogenicity, DNA exhibits excellent biocompatibility and can be safely degraded *in vivo*. Collectively, the unique structural, functional, and translational attributes of DNA hydrogels closely align with the core demands of regenerative medicine, positioning them as a highly promising and versatile platform in clinical potential.

Several recent reviews have comprehensively summarized specific aspects of DNA hydrogel research, including molecular design and tissue engineering applications, musculoskeletal regeneration, biomedical engineering, and general regenerative medicine strategies [[10], [11], [12]]. While these contributions have significantly advanced the field, most focus on either individual application domains, or specific therapeutic indications. In contrast, the present review systematically examines DNA hydrogels from molecular design and chemical synthesis to multiscale biological functions and translational potential in tissue regeneration. We also emphasize intrinsic programmability and dynamic sequence control as design principles, while adopting a clinically oriented perspective. We critically evaluate preclinical evidence and translational progress across regenerative contexts, including bone, cartilage, skin, nerve, cardiovascular regeneration and cancer therapy. By integrating considerations of biocompatibility, mechanical adaptability, and stimulus responsiveness, this review may provide some important information for designing DNA-based hydrogels towards regenerative medicine and personalized therapies.

## 2 Molecular properties and design consideration of DNA hydrogels

**Fig. 2:**
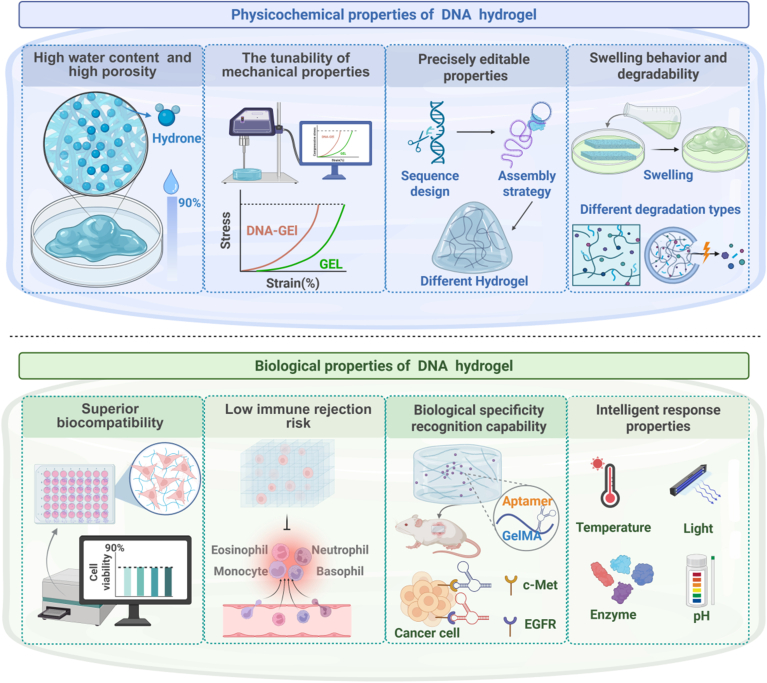
Physicochemical properties and biological properties of DNA hydrogel. Physicochemical properties include high water content and porosity, structural editability, tunable mechanical properties, controllable swelling and degradation and biological properties comprise of superior biocompatibility, low immunogenicity, biospecific recognition, and intelligent responsiveness. This figure was created in BioRender.com.

DNA hydrogels are 3D network hydrophilic polymers constructed with DNA-based pieces. They can combine the biological specificity and editability of DNA with the high water content, biomimetic microstructures, and mechanical flexibility of hydrogels, representing intelligent biomimetic materials (Fig. 2) [13]. Their characteristics determine their potential applications in some fields, such as biomedicine, biosensing, and tissue engineering.

### 2.1 Physicochemical properties

#### 2.1.1 High water content and hydrophilicity

One of the most prominent structural features of DNA hydrogels is the highly hydrated 3D networks with water content typically exceeding 90%, which that closely matches the physiological water content of soft tissues and ECM in living organisms [14]. The high water content not only endows the DNA hydrogels with excellent swelling properties but also provides a near-natural microenvironment for the diffusion of bioactive molecules and cell adhesion. DNA hydrophilicity is determined by multiple interactions at molecular level. The densely distributed phosphate groups in DNA scaffolds carry negative charges, enabling electrostatic adsorption of water molecules to form stable hydration layers. The abundant hydrogen bond donors and acceptors in DNA molecules (amino groups in bases, carbonyl groups, and hydroxyl groups) can form extensive hydrogen bond networks with water molecules, further enhancing hydration stability [15]. The overall negatively charged surface also confers regulatory affinity for positively charged biomolecules (certain drugs and proteins), facilitating functional molecule loading and controlled release. In terms of bio-interface interactions, the synergistic effect of the hydrophilicity and surface charge endows DNA hydrogel with superior adhesion capabilities. DNA hydrogel forms stable and moderate adhesion to damaged tissues through non-covalent interactions of hydrogen bonds and electrostatic forces [16]. This adhesion mechanism ensures interface integrity while avoiding mechanical mismatch or interfacial liquid accumulation, aligning with regenerative microenvironment.

#### 2.1.2 Precisely editable structure and dimensions

Distinguished from traditional hydrogels, DNA hydrogels can achieve the structural programmability. The programmable nature of DNA hydrogels is primarily demonstrated by the ability to precisely design base sequences to predict their assembly and response behavior. The longer the sticky-end sequence of a DNA strand and the higher its G-C content, the greater the binding energy with its complementary strand. This binding energy determines the robustness of cross-linking points in the hydrogel network and reflects its mechanical rigidity and stability [17]. Specific base sequences not only pair but also fold where C-rich sequences fold into i-motif structures under acidic conditions, while G-rich sequences form G-quadruplexes in the presence of potassium ions [18]. The incorporation of these sequences confers pH-responsive and ion-responsive properties to the hydrogel. Techniques such as rolling circle amplification enable the fabrication of long-chain scaffolds with repetitive sequences, which inherently possess regular recognition sites that serve as physical frameworks for three-dimensional hydrogel networks [19]. Building upon sequence encoding, structural programmability allows DNA hydrogels to be customized, ranging from X-shaped and Y-shaped branching units to arbitrary nanostructures resembling origami-like DNA configurations, up to three-dimensional frameworks designed through cross-linking strategies.

Static tunability refers to the ability to continuously and conveniently regulate the macroscopic properties of DNA hydrogels by adjusting formulation parameters (such as DNA concentration and crosslinking agent ratios) during preparation, without altering their molecular coding sequences. Among these parameters, DNA concentration serves as the primary regulatory factor. Higher concentrations can enhance the elastic modulus and mechanical stiffness of hydrogel, thereby influencing molecular recognition efficiency and drug loading capacity [20]. Conversely, lower concentrations yield softer networks with improved diffusivity. Adjusting crosslinking agent ratios further refines crosslink density control, increasing the relative amounts of DNA crosslinkers or chemical agents lead to denser network nodes, resulting in harder hydrogels with reduced swelling, whereas decreasing crosslinking agents improves material flexibility and permeability [21]. Notably, while elevated crosslink density or concentration enhances structural stability, it may hinder substance diffusion and cell migration, compromising sensing sensitivity or tissue regeneration requirements. Furthermore, new technologies such as microfabrication technologies of photolithography and 3D printing techniques enable DNA hydrogels into various morphological structures, including films, fibers, and micropatterns for injectable delivery system, tissue patch, and cell chip platform [22]**.**

#### 2.1.3 Optimal degradability

DNA hydrogels form porous 3D matrices through interconnection of DNA strands, a key feature supporting various biological functions. The porous structure facilitates the transport of essential nutrients and clearance of metabolic waste, thereby creating an optimal microenvironment for tissue regeneration. Additionally, it serves as a conduit for cell migration and provides a supportive scaffold for cell adhesion [23,24]. The pore size of hydrogels determines the diffusion rate of biomolecules (such as proteins and drugs) as well as cell adhesion and proliferation behavior. At the molecular level, hydrogel pore size influences force transmission regulated by cell membrane proteins, cytoskeletal remodeling, organelle distribution, nuclear membrane protein activity, and chromatin reorganization. By activating various mechanotransduction signals, all these changes ultimately affect cellular morphology [25].

Hydrogel swelling is correlated with the hydrophilicity and pore size of the 3D network, which can be regulated by altering the base composition of DNA sequences and crosslinking density to modulate swelling behavior in solutions with varying pH and ionic strength [26]. The degradation capability of DNA crosslinking networks depends on their network structure and crosslinking density. Specific enzymatic degradation can be achieved using nucleases (DNase I) or restriction endonucleases to generate deoxyribonucleotides. Alternatively, base complementarity dissociation can be regulated by melting temperature to induce physical disintegration. The final degradation products are deoxyribonucleotides, which can be metabolized by organisms without residual toxicity [27]. Therefore, pore size is controlled by adjusting the length of DNA strands and crosslinking density to meet various application requirements during construction.

### 2.2 Biological properties of DNA hydrogels

#### 2.2.1 Superior biocompatibility

DNA exhibits excellent compatibility with biomolecules, cells, and human tissues, without inducing strong inflammation reaction [28]. The fundamental building block of DNA, deoxyribonucleotides, are endogenous substances in the human body. Products (nucleotides and phosphates), generated during hydrogel degradation can be cleared or reused through normal metabolic pathways, fundamentally avoiding the metabolic toxicity issues. The high water content of DNA hydrogels and their structural similarity to extracellular matrices reduce mechanical friction between materials and tissues, enabling adaptation to the complex physiological microenvironment *in vivo*. This facilitates cell adhesion, proliferation, and tissue integration. In the single-cell encapsulation level, DNA framework structures serve as nucleating agents to induce the formation of DNA hydrogel layers, which provide long-term stable protection for cells. Compared to unencapsulated cells, mechanically stressed encapsulated cells exhibit significantly reduced autophagy levels, indicating that DNA gel encapsulation effectively resists external mechanical damage [29]. The DyNAtrix dynamic DNA crosslinking matrix has demonstrated high biocompatibility in cultivation experiments across multiple cell types, including human mesenchymal stromal cells, pluripotent stem cells, and human trophoblast organoids [30]. Subcutaneous injection of the hydrogel shows no toxicity or inflammatory reaction in H&E staining, complete blood count, or hemolysis tests, confirming its excellent biocompatibility [31].

Compared with widely used natural polymer hydrogels such as collagen and hyaluronic acid (HA), DNA hydrogels retain equivalent excellent intrinsic biocompatibility and non-toxic degradation characteristics. Differently, collagen hydrogels are prone to rapid enzymatic degradation and batch-to-batch variation derived from animal sources, while HA hydrogels lack flexible structural modification capability. Synthetic hydrogels including GelMA and polyethylene glycol (PEG) generally require chemical crosslinkers or photoinitiators during fabrication, which may introduce residual cytotoxicity. By contrast, DNA hydrogels can be assembled under mild aqueous conditions without toxic additives, further expanding their advantages for cell encapsulation and *in vivo* implantation.

#### 2.2.2 Low immune rejection risk

As endogenous genetic substances, natural DNA exhibits negligible immunogenicity under physiological conditions. Notably, synthetic DNA assembled into hydrogel networks presents an even lower risk of immune responses, which is well documented in relevant studies [32,33]. Their surface, rich in negative charges and hydration layers, reduces nonspecific protein adsorption and inhibits the initial stages of immune activation. During *in vivo* degradation, DNA hydrogels are progressively broken down by nucleases into natural physiological metabolites, such as nucleotides, phosphate, and deoxyribose, without generating significant amounts of pro-inflammatory acidic byproducts (e.g., lactic acid) [34]. Under normal physiological conditions, this degradation process does not induce antibody production against the hydrogel itself nor activate the complement system. Artificially synthesized DNA hydrogels can avoid introducing immunostimulatory motifs (unmethylated CpG sequences), thereby preventing activation of innate immune recognition pathways, like Toll-like receptors [33].

The immunomodulatory function of DNA hydrogels has evolved from their inherent low immunogenicity to active remodeling of the immune microenvironment through multi-level regulatory approaches represented by molecular sequence embedding and nanostructure design. At the molecular level, researchers have achieved dual regulation by designing immunosuppressive pure DNA hydrogels (Is-pDNAgels), whose high-density negatively charged framework efficiently removes free chemokines and inhibits excessive infiltration of neutrophils and macrophages. Concurrently, the embedded immunosuppressive oligodeoxynucleotide (Is-ODN) sequences can penetrate activated immune cells, suppressing multiple inflammatory signaling pathways and inhibiting the secretion of various pro-inflammatory cytokines at the transcriptional level, thereby forming a synergistic anti-inflammatory positive feedback loop [35]. Additionally, DNA hydrogels serve as precise platforms for immune checkpoint modulation; for example, delivery of multivalent aptamer-lysosome-targeted chimerics (LYTAC) analogs induces degradation of PD-L1 protein on tumor cell surfaces while simultaneous delivery of siRNA silences PD-L1 gene expression, achieving dual immune checkpoint inhibition via degradation-silencing and effectively activating antitumor immune responses [36]. At the nanoscale level, hydrogels constructed based on tetrahedral framework nucleic acids (tFNA) inherently possess immunomodulatory activity, significantly reducing the expression levels of various pro-inflammatory cytokines and chemokines in macrophages activated by lipopolysaccharide (LPS). The further developed mineralized DNA hydrogel (Cap-gel) can actively promote the polarization of macrophages toward an M2 reparative phenotype by presenting immunomodulatory ligands, thereby achieving synergistic effects of immunomodulation and tissue regeneration [37].

#### 2.2.3 Biological specificity recognition

Relying on the base complementary pairing characteristics of DNA, specifically the precise recognition between adenine (A) and thymine (T), as well as guanine (G) and cytosine (C), DNA hydrogels can achieve specific recognition and binding to target DNA/RNA sequences. Based on bioengineering modifications of DNA strands (conjugation with antibodies, aptamers, polypeptides, or glycan chains), they enable precise recognition of biological targets, such as small molecules, proteins, and cells, endowing DNA materials with targeting capability [38]. For instance, aptamer-functionalized DNA hydrogels can target specific cell membrane receptors for cellular-specific capture and precise drug delivery [39]. Stimulus-responsive hydrogels based on complementary sequence hybridization can undergo conformational changes or network collapse upon detecting specific nucleic acid signals, enabling controlled release or biosensing. This sequence-encoded recognition mechanism exhibits high programmability, recognizing that different targets can be achieved simply by modifying the sequence design without redesigning the material framework [40]. Consequently, DNA hydrogels not only mimic ligand-receptor interactions in ECM but also serve as intelligent response platform, enabling a functional leap from passive carriers to active recognition for precision medicine, biosensing, targeted delivery, and tissue engineering.

#### 2.2.4 Intelligent response properties

DNA conformation is susceptible to environmental stimuli, which can induce swelling, contraction, degradation, or morphological changes in the hydrogel's 3D network, endowing DNA hydrogels with stimulus responsiveness and classifying them as smart hydrogels. Nucleic acid sequence-responsive DNA hydrogels induce conformational changes through nucleic acid aptamer-target specific binding to regulate the hydrogel network [41]. Environmentally physical stimulus-responsive DNA hydrogels enable targeted dissociation in specific regions by responding to physical signals, such as temperature, pH, ionic strength, and light [42]. Bioenzyme-responsive DNA hydrogels can be cleavaged by specific nucleases (DNase I, restriction endonucleases) or proteases (peptide crosslinking chains), achieving site-specific gel degradation [43]. Additionally, small molecule-responsive DNA hydrogels undergo conformational changes through partial DNA sequences binding to specific small molecules (e.g., ATP), thereby modulating the gel network structure [44].

The responsiveness of DNA hydrogels represents their core advantage as smart materials. Response types must be engineered around specific trigger signals to ensure specificity, sensitivity, and reversibility. For instance, in tumor-targeted therapy, tumor microenvironment-specific signals can be selected as triggers to enable targeted drug release at tumor sites while minimizing damage to normal tissues [45]. A biosensor for detecting low-concentration target substances can identify target molecules through DNA amplification in DNA hydrogels, thereby obtaining higher concentrations of DNA reaction units [46]. In scenarios requiring reusability, reversibly responsive DNA hydrogels should be designed for reusable biosensors. A novel DNA-polymer cross-linked hydrogels has been prepared base on the reversible Schiff base reactions between oxidized polysaccharides and DNA's amino groups through mild aqueous-phase reactions. This process requires neither organic solvents nor high-energy conditions, with the resulting product exhibiting multi-loop recycling characteristics [47].

## 3 Construction strategies and classification of DNA hydrogels

**Fig. 3:**
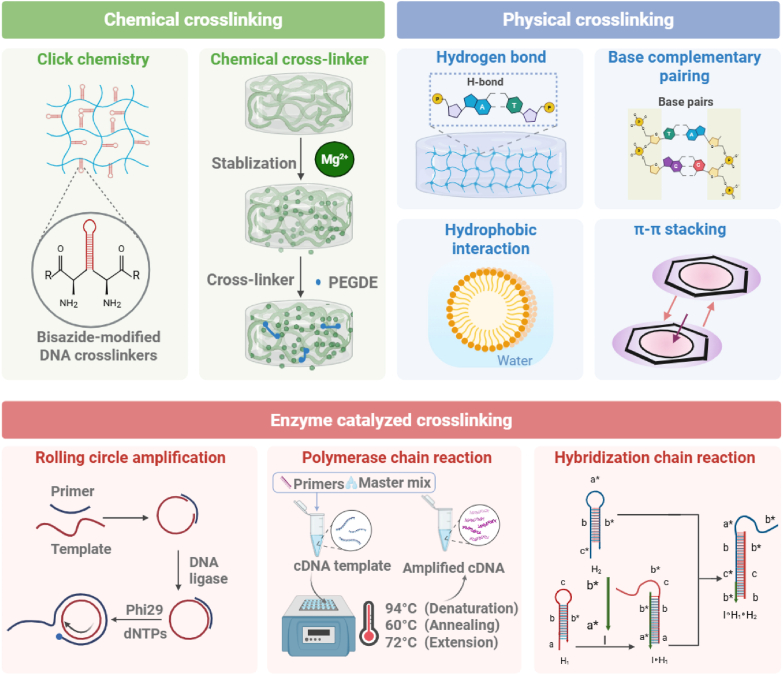
Crosslinking mechanism of DNA hydrogel. Chemical crosslinking includes click chemistry and chemical cross-linker. Physical crosslinking includes hydrogen bond, base complementary pairing, hydrophobic interaction and π-π stacking. And Enzyme catalyzed crosslinking includes rolling circle amplification, polymerase chain reaction and hybridization chain reaction. This figure was created in BioRender.com.

Based on compositional differences, the classification can be defined into pure and hybrid DNA hydrogels. Pure DNA hydrogels consist of DNA as the structural component, forming a 3D hydrophilic network system through base complementarity pairing, enzyme-catalyzed ligation, or long-chain entanglement. Typical building blocks include branched DNA modules, such as Y-shaped and X-shaped structures. However, pure DNA hydrogels often exhibit low mechanical strength. To solve this problem, hybrid DNA hydrogels are composite hydrogel systems that incorporate DNA with non-nucleic acid components, such as natural/synthetic polymers, inorganic nanomaterials, and bioactive molecules [48]. Common composite matrices include polyethylene glycol, gelatin, chitosan, gold nanoparticles, and peptides. These hydrogels not only retain the programmability of DNA, but also allow flexible modulation of macroscopic properties, such as mechanical strength, stability, and adhesion, addressing the limitations of pure DNA hydrogels in terms of mechanical performance and formability. This represents the mainstream direction for practical development of DNA hydrogels (Fig. 3).

In comparison with conventional hydrogel systems, the most prominent advantage of DNA-based hydrogels lies in sequence-dependent structural programmability. Traditional collagen, chitosan and PEG hydrogels rely on physical blending or random chemical crosslinking, whose network structure and functional sites can only be adjusted empirically. In contrast, DNA hydrogels enable precise, molecular-level customization of network topology, pore size, crosslinking density and functional distribution via rational DNA sequence design, which cannot be achieved by most conventional hydrogel materials.

### 3.1 Pure DNA hydrogel construction

The construction of pure DNA hydrogels primarily relies on the chemical properties and interactions of DNA molecules themselves. Their core advantages lie in excellent biocompatibility and precise structural programmability. When DNA strands are in a suitable physiological environment, complementary sequence regions form double-stranded structures through hydrogen bonding and base stacking interaction. These double-stranded structures serve as physical crosslinking points, connecting dispersed DNA molecules to gradually form a 3D gel network. Physical crosslinking constructs networks through non-covalent interactions without forming chemical covalent bonds, exhibiting strong reversibility and mild preparation conditions, typically achieved via self-assembly method. Self-assembled DNA hydrogels mainly utilize branched DNA building blocks with complementary viscous termini (X-DNA, three-arm DNA, and Y-shaped scaffolds) to form 3D networks through specific interaction. For example, Y-shaped scaffolds rapidly self-assemble with linker DNA through terminal hybridization, with strength regulated by terminal length and matching degree, and restriction enzyme cleavage sites can be introduced for enzyme-triggered degradation [49]. In contrast, chemical crosslinking forms irreversible chemical covalent bonds to construct 3D networks, endowing hydrogels with high stability and mechanical strength. This represents a critical construction approach for achieving long-term *in vivo* applications of pure DNA hydrogels. Salmon sperm DNA is utilized to prepare chemically crosslinked linear DNA hydrogels [50]. Another method of chemical crosslinking is enzymatic crosslinking, which utilizes DNA-specific ligases (T4 DNA ligase, DNA ligase I) as catalytic mediators. These enzymes recognize specific structures at the ends of DNA fragments and catalyze the formation of phosphate diester bonds between adjacent DNA molecules 5′-phosphate groups and 3′-hydroxyl groups, gradually linking dispersed DNA fragments into continuous long chains or cross-linked network. Designed complementary sticky-end DNA fragments can be mixed with T4 DNA ligase and specifically catalyzed to covalently bind with complementary DNA fragments under conditions of 37^°^C and Mg^2+^ presence, forming crosslinking nodes to construct three-dimensional gel networks [51]. Chemical modifications can introduce click chemistry-specific reactive groups at the ends or side chains of DNA strands, such as azide groups combined with alkyne groups [52], or thiol groups paired with maleimide groups [53]. The modified DNA molecules are dissolved in a buffer solution, where click chemistry groups undergo specific reactions to form stable covalent bonds, enabling cross-linking between DNA molecules to construct a 3D network. However, chemically cross-linked DNA hydrogels exhibit some limitations. The irreversible covalent bonds between DNA strands create permanent cross-linked structures that lack shear-thinning behavior, rendering them unsuitable for minimally invasive injection applications. These hydrogels lack self-healing properties, restricting their ability to adapt to irregular defects and making them more susceptible to mechanical stress [54].

The high-efficiency amplification properties of nucleic acid amplification techniques can also be utilized. Using short-chain DNA as templates or primers, ultra-long DNA strands or DNA nanostructures with specific topological structures can be amplified in reaction systems containing polymerases and dNTPs (deoxyribonucleoside triphosphates). These amplified products spontaneously form 3D gel network through interstrand entanglement, local complementary pairing, or physical cross-linking interactions. Key amplification techniques include rolling circle amplification (RCA), hybridization chain reaction (HCR), and PCR derivative techniques [55]. Hydrogel preparation typically employs RCA reaction, which requires initial circular DNA synthesis but suffers from low raw material utilization rates and necessitate precision temperature control equipment, thereby increasing operational complexity and cost.

### 3.2 Hybrid DNA hydrogel construction

Hybrid DNA hydrogels overcome the performance limitations of pure DNA hydrogels by incorporating exogenous materials, thereby balancing the functional designability of DNA with the superior properties of exogenous materials and expanding application scenarios. The introduction of polymers significantly enhances the mechanical properties, stability, and degradation control capabilities of hydrogels [56]. Based on polymer sources, hybrid hydrogels can be classified into two categories: natural polymer composites and synthetic polymer composites. Natural polymers include biologically derived macromolecular materials, such as chitosan, hyaluronic acid (HA), and collagen, which inherently possess excellent biocompatibility and bioactivity. Synthetic polymers primarily consist of structurally defined and performance-controlled synthetic macromolecular materials, including polyacrylamide (PAM), polyethylene glycol (PEG), and polylactic acid (PLA). Considering the biocompatibility of natural polymers and the functional designability of DNA, blending and cross-linking approaches can be employed. Chitosan, a positively charged linear polysaccharide, forms composite hydrogels through strong electrostatic interactions with DNA [57].

Tannic acid (TA), a molecule widely present in many plants, utilizes its abundant hydrogen bonds to interact with the phosphate backbone of DNA, forming TA + DNA (TNA) hydrogels. The hydrolyzable ester bonds connecting catechol and pyroglucoside groups in TA confer unique degradation properties to the gel [58]. DNA oligonucleotides and type I collagen can also trigger rapid fiber formation and spontaneous assembly through electrostatic interaction, resulting in nucleic acid-collagen complexes (NACCs) hydrogels. The elasticity of these hydrogels can be regulated by the molar ratio of collagen to DNA, ssDNA length, and collagen type [59]. DNA molecules can be linked to synthetic polymer chains via covalent or non-covalent bonds to form composite gels of DNA-synthetic polymers. Utilizing the temperature sensitivity and sequence-specific complementary pairing of DNA double strands as dynamic crosslinking bonds, two star-shaped polymer-DNA precursors are prepared with complementary sequences. The mixed solutions of these precursors remain in liquid phase at high temperature and form gels at low temperature, corresponding to the dissociation of DNA double strands and the binding of DNA chains respectively [60]. Building upon dynamic non-covalent crosslinking, isocyanate-terminated star-shaped polymers are further introduced as covalent crosslinking agents. Denatured DNA molecules are utilized as building blocks to construct thermoresponsive hydrogels by crosslinking DNA strands with isocyanate (NCO) terminal cap star-shaped poly(EO-stat-PO) crosslinkers. The NCO groups react with the amino groups in DNA bases through urea chains, while base pairing between complementary DNA strands enables DNA self-assembly into a 3D hydrogel network [61]. The dynamic crosslinking approach can also be extended to multi-component system, where electron donors (dopamine) are modified onto the hydroxyethyl methylcellulose (CMC) backbone by introducing self-hybridizable G-quadruplexes. A multi-responsive hydrogel is constructed through dual crosslinking via non-covalent donor-acceptor interaction between dithiophene ethyne and dopamine, coupled with hybridization of G-quadruplexes [62].

Nanomaterials possess unique physicochemical properties (mechanical reinforcement, optical characteristics, and magnetic response). When combined with DNA, they can endow DNA hydrogels with novel functionalities while enhancing their mechanical properties and stability [63]. Metal-based nanomaterials include gold nanoparticles (AuNPs) and magnetic NPs (Fe_3_O_4_, CoFe_2_O_4_). The conventional approach involves first surface-functionalizing metal-based NPs, followed by leveraging the specific interaction between these groups and DNA molecules to anchor DNA onto the surface of NPs. Subsequently, DNA-loaded NPs are interconnected through base complementarity pairing or cross-linking interaction, forming a 3D gel network with NPs serving as cross-linking nodes. The surface of synthetic Fe_3_O_4_@nSiO_2_@mSiO_2_ NPs is modified via surface amination, and mesopores are utilized to load a large amount of luminescent dyes. Nucleic acid initiation units are introduced onto the prepared NPs. These units induce pre-designed, modified polyacrylamide DNA chains to undergo hybridization chain reaction on the NP surface, resulting in a DNA-crosslinked hydrogel coating. DNA cross-linked hydrogels enable targeted controlled release of NPs [64]. Inorganic NPs include graphene oxide (GO), carbon nanotubes (CNT), hydroxyapatite (HAp), and silica (SiO_2_) NPs, among other inorganic non-metallic nanomaterials. Through physical adsorption, chemical modification, or specific interactions, DNA molecules are conjugated with inorganic NPs, followed by the utilization of DNA cross-linking or self-aggregation effects of inorganic NPs to construct composite gel network. DNA-encapsulated carbon nanotube (CNT) composite (DNA-CNT) gels have been successfully fabricated via single-step lyophilization cooling. DNA-encapsulated CNTs form surface helical encapsulation through non-covalent π-π stacking interaction between DNA nucleobases and CNT walls, resulting in water-soluble molecular complexes with highly porous, randomly branched, and low-density structures [65]. Quantum dots (QDs), including semiconductor QDs (CdTe, CdSe, and ZnS) exhibit unique fluorescence properties. For instance, MXene nanosheets doped with MoS_2_ QDs are encapsulated within a DNA gel matrix prepared through 3D printing technology, where salmon sperm DNA is photoligated with polyethylene glycol diacrylate (PEGDA). The prepared composite hydrogel exhibits a porous network structure, which facilitates cargo encapsulation and enables controlled release [66]. MoS_2_-doped MXene (MXMoS_2_) enhances photothermal conversion by improving near-infrared absorption and non-radiative relaxation. However, the integration of QDs into hydrogels through chemical conjugation, entrapment, and polymerization involves complex steps and additional reagents, potentially introducing variability in the final construct composition. DNA complementarity has been utilized to incorporate DNA template QDs with high quantum yield, long-term photostability, and low cytotoxicity into hydrogel network via one-step self-assembly, forming QD-DNA hydrogels (QDH) under physiological conditions [67].

Controllable construction of hydrogel structures through advanced manufacturing techniques enables precise matching of structure and function without directly forming hydrogel networks. This approach achieves macroscopic morphology, microscopic structure, or dynamic response properties of hydrogels, serving as a critical supporting technology for practical application of DNA hydrogels. 3D printing technology utilizes DNA hydrogel precursors (incompletely cross-linked DNA solution or DNA-polymer composite precursor) as printing inks, employing the layer-by-layer accumulation principle of 3D printing to precisely construct 3D macrostructures [68]. Preparation of high-viscosity DNA inks has demonstrated shear-thinning behavior by dissolving 4-5% (w/v) salmon sperm DNA in PBS buffer. Subsequently, these inks are extruded using a custom-made 3D printer via pneumatic extrusion. The structures are immersed in a solution containing acrylamide (AA) and an initiator for post-processing via FASIE, successfully yielding cubic, star-shaped, and other micron-scale DNA hydrogel 3D structures [69]. DNA-induced biomineralization can integrate DNA and biosilica into sodium alginate-based hydrogels to form functionalized inks. Machine learning is employed to optimize 3D printing parameters for high-precision customized printing, while ionic crosslinking (CaCl_2_) stabilizes the printed structures, ultimately yielding 3D-printed hydrogel dressings with excellent porosity, mechanical tunability, and bioactivity [70].

Photopatterning technology utilizes light-responsive DNA molecules (DNA modified with photosensitive groups or containing light-responsive bases) or photocrosslinkers, combined with optical techniques like photolithography and photomasks, to precisely control the crosslinking regions of hydrogels in space, enabling micro-patterned construction [71]. A DNA-based intelligent hydrogel preparation strategy based on photopatterning employs polyacrylamide as the scaffold, embedded with photosensitive DNA modules and modified with o-nitrophenyl phosphate esters. Precise illumination through mask lithography or two-photon laser scanning technology generates periodically distributed circular pattern regions. The prepared hydrogels utilize DNA strand substitution reaction to introduce DNA sequences for specific interaction with target cell membrane surfaces into different DNA patterned areas, regulating cell growth. This achieves spatiotemporally controllable patterning of light-responsive DNA hydrogels and modulation of biophysical and chemical properties to achieve localized cellular responsiveness [72]. Light-responsive DNA hydrogels can be synthesized by preparing double-stranded DNA crosslinkers, and mixing them with acrylamide, chemical crosslinkers, and photoinitiators to form a pre-gel solution. After injection into molds, the solution is shaped through a digital micromirror device system under 365 nm UV light irradiation for 10 s. The resulting gel exhibits a simple structure, with its complex deformation capability derived from programmable light-controlled modulation of DNA crosslinking bonds [73].

## 4 Role of DNA hydrogels in tissue engineering

### 4.1 Cell culture and 3D cell culture

DNA hydrogels exploit Watson-Crick base pairing to achieve molecular-level precision in the regulation of biochemical and biophysical cues, enabling the recapitulation of key features in native ECM and establishing them as powerful platforms for 3D cell culture and functional tissue construction. However, their pore size, crosslinking density, and mechanical strength are predominantly regulated through DNA sequence design and concentration, offering limited tuning dimensions and constraining their ability to mimic the multiscale heterogeneity of native ECM. To overcome these limitations, rolling circle amplification (RCA)-derived products have been leveraged to construct flexible scaffold networks. By systematically modulating RCA reaction time, scaffold concentration, and geometric configuration, DNA hydrogels have been developed with controllable microstructures, enhanced mechanical strength, and precise regulation of cell migration [74]. Such hydrogels exhibit improved biocompatibility and mechanical robustness while enabling fine control over cellular motility through rational microstructural design.

**Fig. 4:**
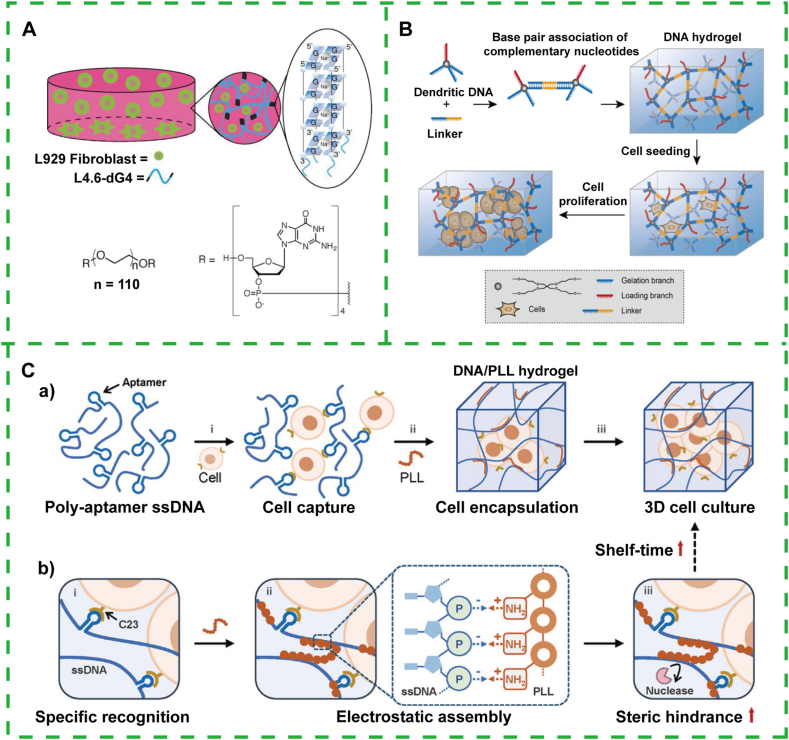
DNA hydrogel in cell culture and 3D tissue construction. (A) Schematic illustration of cell culture on/in G-quadruplex hydrogels. Wavy blue lines represent PEG segments, and chemical structure of L4.6k-dG4 [75]. Copyright 2019, MDPI. (B) Self-assembly of the dendritic DNA hydrogel (DDH) for 3D cell culture [76]. Copyright 2021, American Chemical Society. (C) Synthesis of DNA/PLL hydrogel for 3D cell culture [77]. Copyright 2024, Wiley. (For interpretation of the references to color in this figure legend, the reader is referred to the Web version of this article.)

An alternative physical crosslinking strategy relies on the reversible assembly of G-quadruplex structures. Poly(ethylene glycol)-oligodeoxynucleotide (PEG-ODN) conjugates undergo Na^+^-induced quadruplex formation to yield physiologically responsive DNA hydrogels (Fig. 4A) [75]. Although some cell types remain spherical inside these networks due to the lack of cell-adhesive ligands, their reversible gelation and sedimentation behaviors highlight their potential as cryopreservation-free cell storage matrices. Future improvements, such as incorporating adhesive components or modulating gel density to prevent cell settling, are expected to enable both 3D cell culture and non-cryogenic cell storage in DNA hydrogel systems.

Dendritic DNA hydrogels (DDHs) achieve isothermal gelation at physiological temperature using only nanomolar concentrations of DNA, forming purely crosslinked network with tunable mechanical strength, reversible thixotropy, and micrometer-scale pore sizes (Fig. 4B) [76]. This strategy eliminates the risk of chemical crosslinker contamination and supports in situ encapsulation of both cancerous and somatic cells, maintaining high proliferative activity and enabling repeated passaging. Moreover, the dendritic architecture facilitates the loading of bioactive molecules, allowing precise regulation of cell fate.

Conventional DNA hydrogels constructed via base complementarity, enzymatic reactions, or chemical crosslinking typically undergo degradation through nuclease-mediated cleavage of phosphodiester backbones or stimulus-induced strand dissociation. At the molecular level, nuclease degradation is commonly governed by Michaelis-Menten-type enzymatic kinetics. However, the apparent degradation behavior of bulk DNA hydrogels is also strongly influenced by enzyme transport within the network. Parameters such as crosslink density, mesh size, and network topology regulate enzyme accessibility to cleavage sites and may shift degradation from reaction-limited to diffusion-limited regimes. In densely crosslinked hydrogels, restricted enzyme penetration can substantially retard degradation, whereas more open networks permit rapid nuclease diffusion and accelerated structural disassembly. To achieve spatiotemporally controlled cell release, DNA/poly(L-lysine) (PLL) hydrogels integrate crosslinking functionality with nuclease-protective coatings, prolonging DNA degradation kinetics by approximately 15-fold. Mechanistically, the positively charged PLL chains electrostatically interact with the negatively charged phosphate backbone of DNA, generating a condensed network structure with reduced effective pore size. This compact architecture not only sterically impedes DNase I penetration but also partially masks enzymatic cleavage sites, thereby decreasing enzyme accessibility and significantly slowing hydrogel degradation. Simultaneous incorporation of poly-AS1411 aptamers enhances cell capture efficiency and supports spheroid formation (Fig. 4C) [77]. Nevertheless, PLL-induced rapid gelation may result in nonspecific cell encapsulation, indicating that further optimization is required to improve target cell purity.

Despite the above advances, achieving an optimal balance between rapid gelation kinetics and long-term structural stability remains a universal challenge for 3D culture platforms. Unmodified DNA hydrogels generally show insufficient mechanical strength and rapid enzymatic degradation, which need to be compensated via hybrid crosslinking or material reinforcement strategies.

### 4.2 Drug delivery

**Fig. 5:**
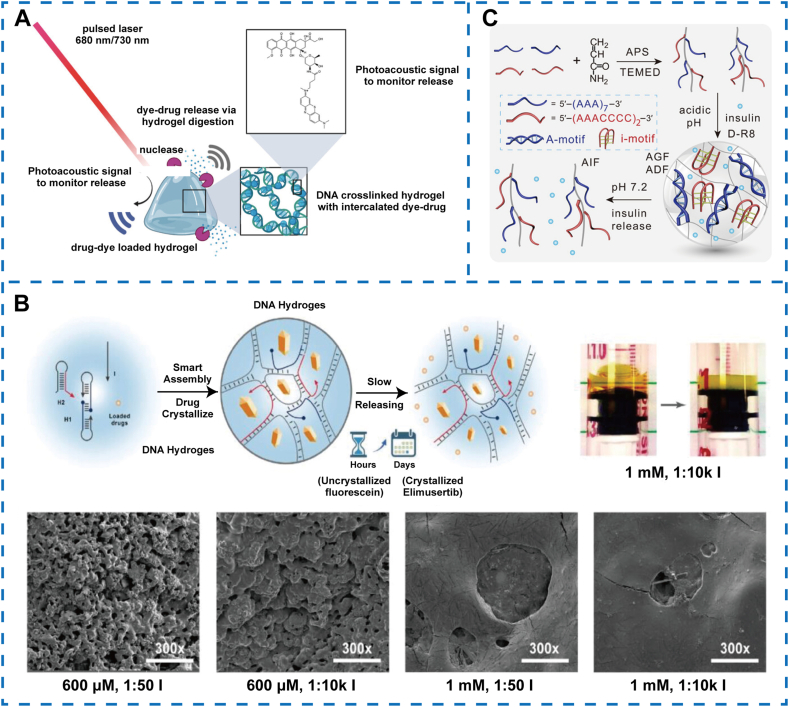
DNA hydrogel in drug delivery. (A) Biodegradable hydrogel photoacoustically monitors chemotherapeutic drug release. Pure DNA cross-linked hydrogel is loaded with methylene blue-doxorubicin (MB-Dox) dye-drug conjugate via hydrophobic binding. MB-Dox has activatable wavelength-specific photoacoustic (PA) signal when loaded in the hydrogel and during drug release [81]. Copyright 2022, Wiley. (B) Encapsulation and sustained release process of Elimusertib in DNA hydrogel, representative photographs of drug-loaded DNA hydrogel before and after gelation in syringe and representative SEM images of drug-loaded hydrogel with a scale of 200 μm [82]. Copyright 2023, Wiley. (C) Schematic illustration for acid-resistant and physiological pH-responsive DNA hydrogel preparation and insulin release [83]. Copyright 2022, American Chemical Society. (For interpretation of the references to color in this figure legend, the reader is referred to the Web version of this article.)

DNA hydrogels, by virtue of their programmable sequences, stimulus responsiveness, and molecular recognition capabilities, enable the precise loading of nucleic acid therapeutics while effectively protecting them from enzymatic degradation [78,79]. Their intrinsically porous architecture and highly crosslinked networks confer a strong capacity for efficient encapsulation of diverse therapeutic cargos, thereby offering distinct advantages over conventional delivery materials. Unlike synthetic polymers, DNA hydrogels exhibit sequence-defined degradation kinetics and can encode multiple release triggers within a single construct, allowing delivery strategies that more closely emulate the dynamic behavior of the native ECM [80]. Notably, supramolecular DNA hydrogels has further overcome limitations in loading efficiency, achieving encapsulation efficiencies exceeding 90% for both nucleic acid therapeutics and small-molecule drugs (Fig. 5A) [81]. These injectable platforms enable real-time monitoring of release dynamics via photoacoustic imaging and afford precise spatiotemporal control without the need for chemical modification of the payloads, while simultaneously avoiding burst release and drug retention issues commonly associated with traditional encapsulation approaches.

Injectable biodegradable hydrogels combine minimally invasive administration with in situ gelation and retention, and their intrinsic degradability eliminates the need for secondary surgical intervention. Moreover, modulation of degradation rates enables sustained local drug release. A graphene oxide (GO)-crosslinked DNA polyaptamer hydrogel constructed via a one-step RCA strategy has been reported to achieve specific loading and injectable delivery of kanamycin [84]. This system integrates aptamer functionality with GO-mediated reinforcement in a single-step process, while the modular aptamer design confers broad platform versatility. Nevertheless, challenges still remain, including suboptimal optimization of loading efficiency and capacity, incomplete quantitative understanding of degradation-release coupling mechanisms, and insufficient systematic evaluation of the long-term biocompatibility of GO nanosheets, their degradation pathways, and overall clinical translatability—factors that currently constrain progression toward clinical application. In the context of combination therapy, DNA hydrogels demonstrate the unique capacity for orthogonal cargo loading. A pH-responsive dendritic nanogel (pH-DNG) developed enables ratiometric co-delivery of small-molecule chemotherapeutics and gene therapeutics, simultaneously encapsulating antisense oligonucleotides (ASOs) and doxorubicin [85]. In a triple-negative breast cancer model, pH-DNG achieves tumor growth inhibition rate of 72%, significantly outperforming monotherapies. This platform establishes a temporally programmed therapeutic window through differential release kinetics: ASOs are rapidly released within 6 h via pH-triggered scaffold disassembly, whereas doxorubicin is released gradually over up to 72 h through sustained diffusion. Similarly, an injectable DNA-RNA hybrid hydrogel achieves precise dual-RNA delivery via independently regulated mechanism, based on enzyme-responsive degradation-mediated release of siRNA and toehold-mediated strand displacement for miRNA delivery [86]. This construct maintains *in vivo* bioactivity for over 14 days, effectively addressing the inherently short half-life of RNA therapeutics.

DNA dendritic polymers can be assembled via a one-step process into nanogels with diameters of several hundred nanometers, enabling dual functionality through the co-delivery of chemotherapeutic agents and nucleic acid aptamers [87,88]. Programmable DNA hydrogel-assisted microcrystalline formulations have been developed for localized sustained drug delivery targeting residual tumor lesions following surgery as well as unresectable lymph node metastases (Fig. 5B) [82]. This system achieves nearly two weeks of sustained release of the ATR inhibitor elimusertib via a microcrystallization mechanism, while smart gelation triggered by pure DNA strands and catalytic reactions allows precise control over mesh size and mechanical properties. To address delivery challenges under specialized physiological conditions, acid-resistant DNA hydrogels have enabled breakthroughs in oral administration. A pH-responsive DNA hydrogel crosslinked by A-motif duplexes (pH 1.2-3.0) and i-motif quadruplexes (pH 4.0-6.0) remains stable in gastric acid to protect insulin while disassembling under physiological pH to release the payload; its oral delivery feasibility has been validated in diabetic rat models (Fig. 5C) [83]. This paradigm of gastric sequestration-intestinal release introduces a new path for oral delivery of protein therapeutics. However, further clarification of DNA-acrylamide copolymerization mechanisms, comprehensive loading capacity data, systematic immunogenicity assessment of degradation products, head-to-head comparisons with existing oral delivery systems, and cost-quality control considerations for scalable manufacturing remain necessary.

DNA hydrogels have evolved from academic curiosities into sophisticated drug delivery platforms capable of addressing critical challenges in regenerative medicine. Recent advances reveal clear trends toward multi-stimuli responsiveness, integrated theranostics, and combination therapy delivery, with key innovations establishing new benchmarks in loading efficiency, controllable release mechanisms, and real-time optical monitoring. The convergence of DNA nanotechnology with advanced manufacturing techniques and computational design is expected to accelerate the realization of clinically deployable, patient-adaptive therapeutic systems. These programmable delivery mechanisms lay the foundation for localized therapy in various tissue repair scenarios.

### 4.3 Biosensing and diagnosis

Owing to their programmable 3D nucleic acid network architectures, DNA hydrogels have emerged as multifunctional platforms with distinct advantages in biosensing and diagnostic applications [89,90]. Beyond serving as passive scaffolds, DNA hydrogels can simultaneously function as signal amplification carriers, reaction regulators, and visualization. They can be seamlessly integrated with isothermal amplification techniques, nanomaterials, and lateral flow chromatography, enabling broad-spectrum detection of nucleic acids, proteins, and small-molecule targets. Consequently, DNA-based hydrogels demonstrate considerable promise for point-of-care clinical diagnostics and safety monitoring, offering high sensitivity, multiplexed detection capability, and portability [[91], [92], [93], [94], [95]].

At the level of signal transduction and amplification, CRISPR-Cas12a-coupled DNA-based fluorescence/colorimetric dual-mode sensors have been developed in which aptamer recognition events initiate catalytic hairpin assembly (CHA) cascades, achieving ultrasensitive detection of both small molecules and proteins [96]. However, current studies don't elucidate how the 3D hydrogel microenvironment enhances CRISPR cleavage kinetics, and the establishment of quantitative correlations between network structural parameters (porosity and crosslinking density) and sensing performance is not explained. This gap limits the distinctive value of DNA hydrogels as enzymatic activity enhancers. Future work may benefit from a functional role-oriented narrative framework and the incorporation of theoretical models describing gelation-enzyme kinetics coupling, thereby strengthening differentiation in the rapidly evolving field of portable diagnostics. To address these limitations, CRISPR-responsive DNA hydrogel cascade amplification systems have been developed, in which target miRNAs trigger strand displacement amplification and CHA reactions to precisely regulate Cas-enzyme-mediated hydrogel phase transitions. This modulates self-electrolytic collision frequency of silver NPs, enabling ultrasensitive, immobilization-free and label-free detection based on collision frequency [97]. This triple-amplification strategy pushes the detection limit of miR-141 to 4.21 aM and demonstrates excellent specificity in serum and cellular samples.

Among the widely adopted isothermal signal amplification technologies integrated with DNA hydrogels, RCA, hybridization chain reaction (HCR) and CRISPR-Cas systems possess unique features and targeted application scenarios [[98], [99], [100]] (see Table 1). RCA relies on circular DNA templates and strand-displacement polymerases to generate ultra-long tandem repeat sequences, featuring ultra-high amplification efficiency, strong anti-interference ability in complex biological samples, and suitability for long-term sustained signal output; it is widely applied in trace nucleic acid detection, exosome analysis and long-term in-situ biosensing [101,102]. HCR proceeds via autonomous cascade hybridization of hairpin probes under isothermal conditions, requiring no enzymes or auxiliary cofactors. It has simple operation, mild reaction conditions and excellent biocompatibility, which makes it ideal for cell imaging, intracellular sensing and portable point-of-care testing (POCT). Different from the two enzyme-free or polymerase-based strategies, CRISPR-Cas amplification combines specific target recognition and collateral cleavage activity, exhibiting extraordinary single-nucleotide resolution and ultrahigh specificity. It is particularly competent for highly specific detection of gene mutations, pathogenic microorganisms and low-abundance disease biomarkers, though its performance is easily affected by reaction buffer and environmental conditions. The rational combination of these three strategies further enables multi-level cascade amplification and multiplex detection on DNA hydrogel platforms.

**Table 1:** Comparison of signal amplification strategies used in DNA hydrogel biosensing.

Strategy	Amplification mechanism	Advantages	Limitations	Typical applications
RCA	Polymerase-mediated isothermal amplification	Very high signal output; long DNA products	Requires enzymes	Ultrasensitive nucleic acid and biomarker detection
HCR	Enzyme-free chain reaction	Simple operation; low background; low cost	Lower amplification efficiency than RCA	Point-of-care testing; portable sensing
CRISPR	Programmable target recognition and collateral cleavage	Extremely high specificity; multiplex potential	More complex system design	Pathogen detection; mutation analysis; precision diagnostics

**Fig. 6:**
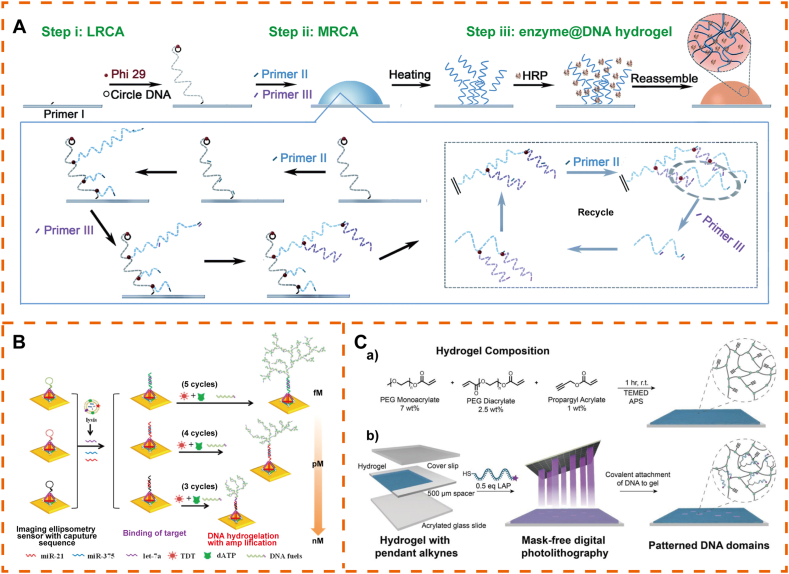
DNA hydrogels in biosensing and diagnosis. (A) Fabrication of the surface-immobilized enzyme@DNA hydrogel [103]. Copyright 2017, Royal Society of Chemistry. (B) Imaging ellipsometry biosensor based on DNA hydrogelation for multiplexed exosomal miRNA detection [104]. Copyright 2020, American Chemical Society. (C) Synthesis of PEG hydrogels with pendant alkyne groups and workflow for photopatterning hydrogels with DNA [105]. Copyright 2023, Wiley.

Concurrently, the role of DNA hydrogels as reaction microenvironment regulators has been further substantiated in enzyme immobilization-based sensing systems. Using a surface-primer-induced strategy, a self-forming, recyclable, pure DNA hydrogel 3D scaffold is constructed on indium tin oxide (ITO) electrodes. By encapsulating enzyme molecules, this platform enables direct colorimetric and electrochemical detection of hydrogen peroxide and bilirubin in serum samples (Fig. 6A) [103]. The molecular sieving effect of the hydrogel effectively excludes macromolecular interferents while permitting free diffusion of small analytes, and the resulting enzyme@DNA hydrogel system exhibits excellent stability and rapid regeneration capability. This approach represents a paradigm shift from conventional 2D interfacial sensing toward fully 3D architectures.

The integration of DNA hydrogels with imaging ellipsometry has further overcome long-standing sensitivity limitations for optical detection. Isothermal amplification mediated by tetrahedral DNA probes generates a high-dielectric-constant 3D network on gold films, reducing the detection limit of exosomal miRNAs. The system exhibits a tunable dynamic range and enables stable multiplex miRNA detection in 50% serum or plasma (Fig. 6B) [104]. Beyond molecular detection, spatial patterning of DNA hydrogels introduces new situations for cell-level diagnostics. Through thiol-yne photochemistry combined with mask-free digital photolithography, micrometer-scale spatial resolution of DNA sequence patterning over centimeter-scale areas is achieved, enabling reversible, sequence-specific protein anchoring via hybridization (Fig. 6C) [105]. This technology allows spatiotemporally controlled activation of localized cellular signaling pathways and enables label-free sensing by monitoring selective cell adhesion and behavioral responses within defined signal domains. Such capabilities lay the foundation for intelligent hydrogels that integrate signal modulation with cell-response-based diagnostics, with potential extension to early detection of multi-signal dysregulation in disease models and high-throughput drug screening. Future efforts should focus on establishing quantitative models linking structural parameters, reaction kinetics, and analytical performance, while advancing modular integration strategies toward clinically viable diagnostic platforms.

Beyond their diagnostic utility, biosensing DNA hydrogels are increasingly evolving towards integrated theranostic platforms that combine molecular recognition, signal transduction, and therapeutic intervention [87]. Disease-associated biomarkers, metabolites, nucleic acids, or microenvironmental cues detected through aptamers, DNAzymes, CRISPR circuits, or amplification networks can serve not only as analytical targets but also as endogenous triggers for drug release, immunomodulation, and regenerative responses. Such coupling of sensing and actuation enables closed-loop regulation, whereby pathological signals are continuously monitored and translated into programmable therapeutic outputs. Consequently, theranostic DNA hydrogels represent a natural extension of biosensing technologies and provide an important bridge between molecular diagnostics and advanced biomedical applications, including precision medicine and tissue regeneration. Beyond independent diagnostic functions, the sensing modules of DNA hydrogels can be integrated with therapeutic units to construct theranostic systems for tissue repair and tumor treatment.

### 4.4 Stimuli-mediated response

Benefiting from the precision of Watson-Crick base pairing, conformational programmability, and excellent biocompatibility, DNA hydrogels have emerged as core platforms for intelligent stimulus-responsive and theranostic materials. Building upon the biosensing mechanisms discussed above, these systems can transform molecular recognition events into programmable therapeutic actions, thereby integrating diagnosis and treatment. They are capable of undergoing reversible sol-gel phase transitions and tunable mechanical modulation in response to endogenous and exogenous stimuli, including pH, temperature, light, and enzymatic activity [[106], [107], [108]]. Although some challenges continue to hinder clinical translation, such as *in vivo* stability, high synthesis cost, and complex logical response construction, the synergistic integration of multiple stimuli has demonstrated substantial potential in controlled drug release, biosensing, and 4D-printed tissue scaffolds [[109], [110], [111], [112]].

**Fig. 7:**
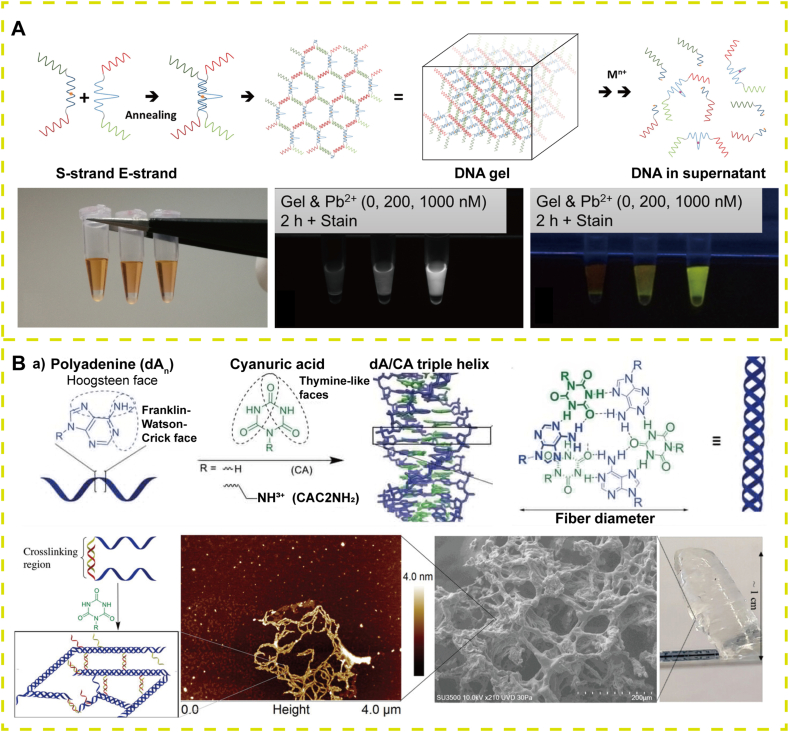
DNA hydrogel in stimuli-mediated response. (A) Preparation of responsive pure DNA hydrogels and principle of metal ion detection. Visualization of DNA hydrogels using nucleic acid dyes: Photographs, color fluorescence images, and grayscale images [117]. Copyright 2021, Elsevier. (B) Representative structures and cross-linking methods of DNA hydrogels. AFM images of diluted hydrogels, SEM images of lyophilized hydrogels, and images of a 4 wt% dA30-dsDNA20 hydrogel sample tilted on a razor blade [118]. Copyright 2023, Wiley. (For interpretation of the references to color in this figure legend, the reader is referred to the Web version of this article.)

DNA hydrogels are increasingly explored as portable platforms for safety inspection and environmental monitoring. For contaminant screening, their 3D networks can be readily functionalized with aptamers and recognition motifs, enabling on-demand integration of diverse signal transduction mechanisms [[113], [114], [115]]. For instance, a core-satellite dual-mode sensor based on DNA-gated metal-organic frameworks (MOFs) and bimetallic nanozymes (Fe_3_O_4_@MOF-gold nanostars) is developed, in which target-induced exponential amplification reactions unlock MOF pores. The released reporter molecule TMB is subsequently catalyzed by the nanozyme, yielding simultaneous colorimetric and SERS signal amplification, with limits of detection for chloramphenicol as low as 2.07 × 10^−8^ M and 7.74 × 10^−12^ M, respectively [116]. This strategy highlights the synergistic amplification enabled by stimulus-responsive DNA nanostructures and nanozymes, and further expansion of aptamer libraries combined with portable readout devices could facilitate commercialization for on-site screening. A Pb^2+^-responsive pure DNA hydrogel integrating a DNAzyme and its substrate strand in a one-step process is reported, forming a label-free sensing platform in which DNAzyme activation induces hydrogel collapse and quantitative release of DNA fragments (Fig. 7A) [117]. While this approach exploits DNA programmability to extend metal-ion sensing with operational simplicity and low cost, improvements in selectivity, mechanical robustness, and lyophilization-enabled portability are still required for the deployment of complex matrices. Addressing the commonly insufficient mechanical strength of DNA hydrogels (typically <10^3^ Pa), a supramolecular poly(deoxyadenosine)-cyanuric acid (poly-dA/CA) fiber system exhibits multi-stimulus responsiveness through small-molecule interactions, sequence recognition, and physiological pH changes. This system combines injectability, self-healing, and shear-thinning properties, and achieves 95% gene silencing efficiency when delivering antisense oligonucleotides (ASOs) under physiological pH, representing a 2–3-fold improvement over free ASOs (Fig. 7B) [118].

Dynamic mechanism design and molecular engineering are central to further performance enhancement. In catalytic cleavage-based systems, dynamically programmed hydrogels construct from DNA circuit architectures and cascade toehold-mediated displacement reactions (TMDR) enable simultaneous catalytic cleavage of crosslinking points and main chains, resulting in sharper and more sensitive gel-sol transitions than conventional non-catalytic systems. Response thresholds can be flexibly tuned via crosslinking density, and functionalization with PEGylated gold NPs as enzyme-free signal amplifiers enables highly sensitive colorimetric ATP detection within 30 min, with a detection limit of 5.6 × 10^−6^ M [119]. Systematic substitution of DNA with charge-neutral morpholino oligonucleotides (MOR) reveals that MOR hydrogels exhibit faster strand-displacement kinetics due to elimination of counterion-driven osmotic pressure effects, although their overall swelling responses are weaker than those of DNA hydrogels [120]. Marked differences in diffusion coefficients, binding/dissociation kinetics, and branch migration rate constants underscore the decisive role of nucleic acid backbone charge states in coupling molecular recognition, mass transport, and macroscopic responsiveness. The versatile stimulus-responsive characteristics enable DNA hydrogels to adapt to complex microenvironments in different tissues, which has been widely applied in targeted tissue regeneration.

### 4.5 Mechanical property

The mechanical performance of DNA hydrogels constitutes a foundational determinant of their biomedical applicability. Key parameters, including storage modulus, pore size, and swelling ratio, are jointly governed by the crosslinking modes, chain length, and topological architecture of the nucleic acid backbone [121]. The correlation analysis is in Table 2. Accordingly, the elucidation and optimization of these mechanical determinants have become central themes in the field. Current research efforts can be broadly categorized into three interrelated dimensions: Fundamental crosslinking mechanisms, composite regulation strategies, and the development of functionally distinctive hydrogel systems. From the perspective of fundamental crosslinking modes, the mechanical behavior of DNA hydrogels is intimately linked to the nature of the crosslinks. Physically crosslinked hydrogels rely on noncovalent interactions, such as hydrogen bonding, base stacking, and higher-order motifs (G-quadruplexes and i-motifs) to form network structures. These materials generally exhibit limited mechanical robustness and are susceptible to environmental stimuli-induced dissociation [122,123]. In contrast, chemically crosslinked DNA hydrogels employ covalent bonds to interconnect nucleic acid to subsequent functionalization [8,[124], [125], [126]].

**Table 2:** Comparison of mechanical properties of DNA hydrogels in different construction strategies.

System type	Construction strategy	Gelation conditions	Storage modulus (G′)	Swelling ratio	Pore size	Advantages	Limitations	Reference
Pure DNA hydrogels	Biomass DNA: Chemical crosslinking	After mixing the DNA solution with PEGDA, place it on a heating plate at 37°C and incubate for 1 h to complete gelation.	1-10 kPa	The swelling process of DNAgel soaked in DW is able to absorb liquid up to 470 times its own weight	Not specified	Wide sources and easy accessibility, exhibits strong and stable adhesion to wet/bloody tissues	Relies on ultra-long chain DNA	50
	DNA nanostar: RCA and click reaction	Hybridize at 30°C for 4 h, perform the click reaction at room temperature for 2 h, and finally incubate at 30°C for 48 h.	10-100Pa	Not specified	Not specified	High mechanical stiffness, excellent stability, and superior sensitivity	The preparation process is highly complex and time-consuming, and sensitive to environmental conditions.	52
	Biomass DNA: RCA	The RCA reaction was performed using DNA polymerase under mild enzymatic conditions	Not specified	Not specified	Not specified	DNA hydrogels can degrade gradually, facilitating cell infiltration and tissue regeneration	The negative charge of DNA may attract positively charged thrombin; in the absence of the NU172 aptamer, this interaction promotes thrombus formation.	53
Hybrid DNA hydrogels	DNA-tannic acid: reversible hydrogen bonds	After the DNA solution was heated to 55°C, TA solutions was added to the DNA solution. The hydrogels were incubated at room temperature for 30 min	>10 kPa	The swelling ratio of TNA gels was measured: ≈6 for [DNA base pair]/[TA] of 0.9 and ≈ 13 for [DNA base pair]/[TA] of 1.3	Not specified	Biodegradability, extensibility, tissue adhesiveness, and hemostatic ability	Expand the scope of application	58
	DNA-type I collagen: self-assembly through electrostatic interaction	Complexation and complete hydrogelation were allowed to proceed at 37°C for 45 min.	Not specified	Not specified	Not specified	Biocompatibility and Shear-thinning behavior of standard syringes during minimally invasive injection	Focus on validating biological functions, without providing the data of storage modulus, swelling behavior, or pore size	59
	DNA-CNTs: self-assembly and freeze-drying	The method was mixing 120 μL HEPES buffer and 1.2 mg CNTs together. Then, 15 μL of 10 mM DNA strand C1 was added. The mixture was sonicated in an ice-water bath for 30 min using a 100 W bath sonicator. In the end, 15 μL of 10 ework for DNA hydrogels in regenemM DNA strand C2 was added, and the final solution was incubated at room temperature overnight and then stored in the fridge at 4°C as the stock.	Not specified	Not specified	The mean height is 21.95 ± 3.23 μm	Three-dimensional structure with high porosity	The mechanical properties require further investigation.	65

**Fig. 8:**
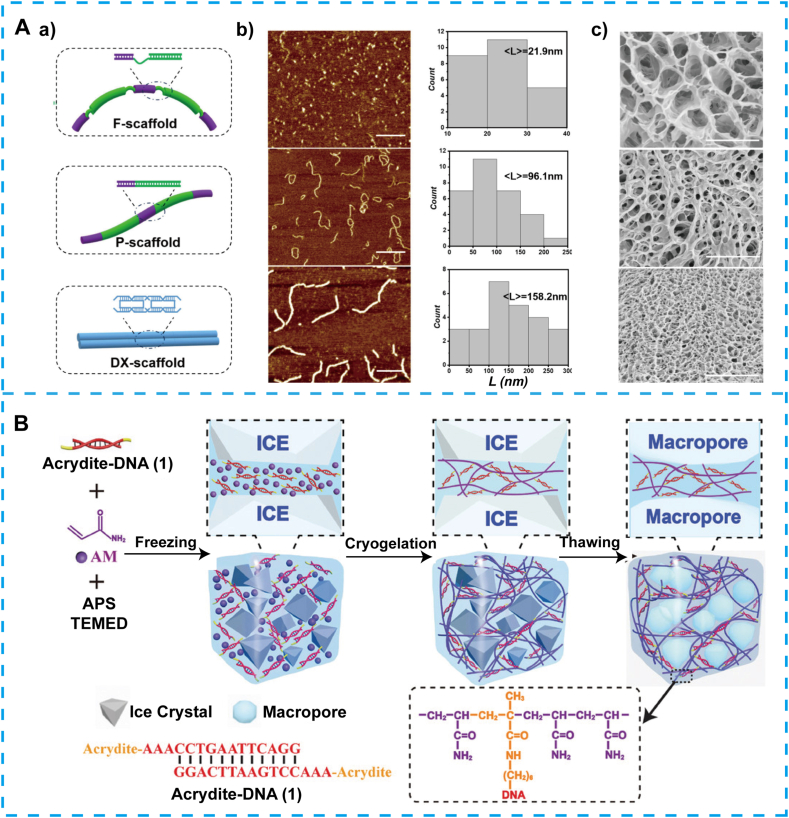
DNA hydrogels in mechanical property. (A) Formation of stiffness tunable DNA hydrogel: Effect of DNA scaffold rigidity on DNA hydrogel stiffness. a) Structures, b) AFM images and c) SEM images, along with length distributions of F scaffolds, P scaffolds, and DX scaffolds [128]. Copyright 2024, American Chemical Society. (B) Schematic representation of the cryostructation process of the pAM-DNA cryogels [129]. Copyright 2020, Wiley.

Synergistic optimization of mechanical performance and functional attributes has been realized through diversified composite strategies. Hybrid crosslinking approaches, incorporation of nanomaterials, and construction of interpenetrating polymer networks effectively overcome the intrinsic limitations associated with single crosslinking modes [127]. Modular design and topological regulation have emerged as powerful tools for tissue regeneration. By tuning the intrinsic stiffness of DNA building blocks and their connectivity patterns, mechanical strength can be systematically modulated while preserving favorable dynamic properties and biocompatibility. Theoretical simulations have further elucidated the kinetics of circular DNA concatenation reactions mediated by DNA topoisomerases, providing rational design guidelines for high-performance DNA gels (Fig. 8A) [128,130]. Additionally, dual rolling circle amplification techniques enable rapid (≤10 s) and facile fabrication of purely physical DNA hydrogels with tunable mechanical properties by modulating the hydrogen-bonding density of ultralong single-stranded DNA precursors. Nevertheless, the precise balance between hydrogen bonding and physical entanglement in such systems remains insufficiently understood and warrants further investigation [131].

From a structure-property perspective, the storage modulus (G′) of DNA hydrogels is primarily governed by the density of elastically effective network chains and the average strand length between neighboring crosslinking junctions [132]. Consistent with classical polymer network elasticity theory, increasing crosslink density generally enhances G′, whereas longer DNA segments between junctions tend to reduce network stiffness due to increased chain flexibility. The modulation of hybridization density, branching valency, and network connectivity can tune G′ over several orders of magnitude, ranging from hundreds of pascals to megapascal levels [133]. Nevertheless, unlike conventional polymer hydrogels, a universal master curve relating crosslink density or strand length to elastic modulus has not yet been established for DNA hydrogels. This limitation arises from the simultaneous contributions of sequence-dependent hybridization thermodynamics, higher-order DNA structures, reversible supramolecular interactions, and network heterogeneity, which collectively complicate quantitative prediction of macroscopic mechanics. In terms of mechanical tunability, conventional hydrogels such as GelMA and PLA realize stiffness adjustment mainly by changing crosslinker concentration or material ratio, with relatively narrow adjustable ranges and single regulation modes. Benefiting from diverse crosslinking modes (physical hybridization, covalent ligation, enzyme catalysis) and topological design, DNA hydrogels achieve a much broader modulus range spanning Pa to MPa. Moreover, their reversible supramolecular interactions endow them with unique viscoelasticity, self-healing and shear-thinning properties, which are superior to many permanently crosslinked synthetic hydrogels and better match the dynamic mechanical microenvironment of native tissues. Such rheological characteristics are increasingly recognized as important regulators of mechanotransduction processes, influencing cell adhesion, migration, lineage specification, and tissue remodeling. Therefore, achievable range of modulus of various tissues, including cartilage, bone, nerve, skin, and heart is shown in Table 3.

**Table 3:** Currently achievable range of G′/E values, the target ranges for various tissues, including cartilage, bone, nerve, skin, and heart.

Tissue	Modulus	Achievable range	Gap assessment
Nerve	0.1 – 1 kPa	Fully covered	Fully covered
Cartilage	10 – 25 kPa	Largely covered	Largely covered
Skin	10 – 100 kPa	Largely covered	Largely covered
heart	10 – 50 kPa	Largely covered	Largely covered
Bone	7 – 30 GPa	Partially covered	Partially covered

With respect to the development of functionally distinctive systems, several DNA hydrogels exhibiting both superior mechanical properties and intelligent responsiveness have been successfully engineered. To address the longstanding challenges of slow mass transport and inadequate mechanical strength, hierarchically structured DNA hydrogels fabricated via freeze-casting leverage ice-crystal template to generate interconnected macroporous networks that accelerate macromolecular diffusion. Concurrently, dense crosslinked regions formed in the unfrozen domains lead to more than an order-of-magnitude increase in storage modulus, enabling the hydrogels to withstand compressive strains of up to 90% without fracture, while improving enzyme-responsive kinetics by over 50% (Fig. 8B) [129]. Further integration with directional freezing polymerization has yielded anisotropic DNA cryogels capable of efficient capture and temperature-controlled release of specific cell populations [134]. Similarly, scanning ultraviolet light-guided assembly induces uniaxial alignment of DNA chains, enabling the construction of patterned anisotropic hydrogels without sequence modification. These materials exhibit pronounced directional dependence in both storage modulus and shape-memory behavior [135]. From the standpoint of biocompatibility optimization, homogeneous hydrogels formed by controlled acidification-induced co-assembly of natural double-stranded DNA and chitosan effectively circumvent rapid phase separation. The derived aerogels and regenerated hydrogels display mechanical strength and elasticity comparable to those of synthetic polyurethane foams, relying exclusively on non-toxic electrostatic interactions [136]. Meanwhile, ATP aptamer-functionalized DNA hydrogels exploit conformational transitions to achieve three-stage, in situ tunable mechanical properties, with storage moduli progressively increasing from 204 Pa to 570 Pa [137]. DNA hydrogels and enantiomeric counterparts have enabled the creation of single-variable stiffness systems that decouple mechanical properties from chemical composition and topological architecture. These platforms reveal a dominant role of degradation-mediated interactions in regulating neural progenitor cell fate, thereby providing an ideal 3D culture system for mechanobiology [138].

Beyond serving as passive structural support, the mechanical properties of DNA hydrogels actively regulate cellular behavior through mechanotransduction mechanisms [139]. Cells perceive matrix stiffness and viscoelasticity via integrin-mediated adhesions, cytoskeletal tension, and force-dependent signaling pathways, which collectively influence cell functions [140,141]. In neural tissue engineering, soft matrices with storage moduli comparable to native brain tissue facilitate neurite extension and neural stem cell differentiation by maintaining low cytoskeletal tension and promoting neurogenic signaling. Conversely, stiffer matrices generally enhance cell spreading, focal adhesion formation, and actomyosin contractility, thereby favoring osteogenic differentiation and extracellular matrix deposition. Importantly, the reversible crosslinking and stress-relaxation characteristics of DNA hydrogels provide dynamic mechanical cues that more closely resemble those of native extracellular matrices than permanently crosslinked synthetic hydrogels. Such viscoelastic regulation has been increasingly recognized as a key determinant of stem cell fate decisions and tissue regeneration outcomes.

The achievable stiffness range of DNA hydrogels should also be considered in the context of specific biomedical applications. For neural tissue engineering, matrices with storage moduli of approximately 0.1-1 kPa are generally favorable for neurite extension and neural lineage commitment, whereas substantially higher stiffness may suppress neurite outgrowth. Notably, the ATP-responsive DNA hydrogel described above, with tunable G′ values between 204 and 570 Pa, falls within this biologically relevant range. In contrast, load-bearing bone tissue engineering typically requires scaffold moduli in the range of several hundred kilopascals to megapascals. Although recent composite and cryogel-based DNA hydrogel systems have approached this stiffness regime, most purely DNA-based hydrogels remain below the mechanical requirements for highly load-bearing osteogenic applications, highlighting the importance of composite reinforcement and multiscale network design. Through optimization of crosslinking modes, integration of composite strategies, topological regulation, and functional molecular modification, DNA hydrogels have achieved broadly tunable mechanical performance spanning biologically relevant stiffness regimes [121]. However, a major remaining challenge is the establishment of predictive quantitative structure-property relationships that correlate crosslink density, strand length, network topology, and dynamic bonding behavior with macroscopic mechanical properties. Such predictive frameworks would facilitate the rational design of DNA hydrogels tailored to the mechanical requirements of specific biomedical applications, ranging from neural regeneration to load-bearing tissue repair.

## 5 Therapeutic potential of DNA hydrogels in tissue regeneration and repair

### 5.1 Bone regeneration and repair

Benefiting from the tunable 3D culture platforms, controllable drug delivery and dynamic stimulus-responsive properties of DNA hydrogels, these materials have become promising interfacial candidates for bone tissue repair [142]. A central challenge in bone regeneration is the temporal mismatch between angiogenesis and mineralization. Accumulating evidence suggests that conventional DNA hydrogels struggle to effectively couple these processes, largely due to an incomplete understanding of DNA-guided biomineralization mechanisms [143,144]. To address this issue, an enzyme-programmable DNA-PEG hydrogel is designed for metalloproteinase-responsive release of vascular endothelial growth factor (VEGF) to initiate angiogenesis, followed by nuclease-catalyzed generation of phosphate ions that drive mineral deposition. This system enables spatiotemporally sequential regulation of angiogenesis, osteogenesis, and mineralization, thereby significantly enhancing bone regeneration outcomes [145].

**Fig. 9:**
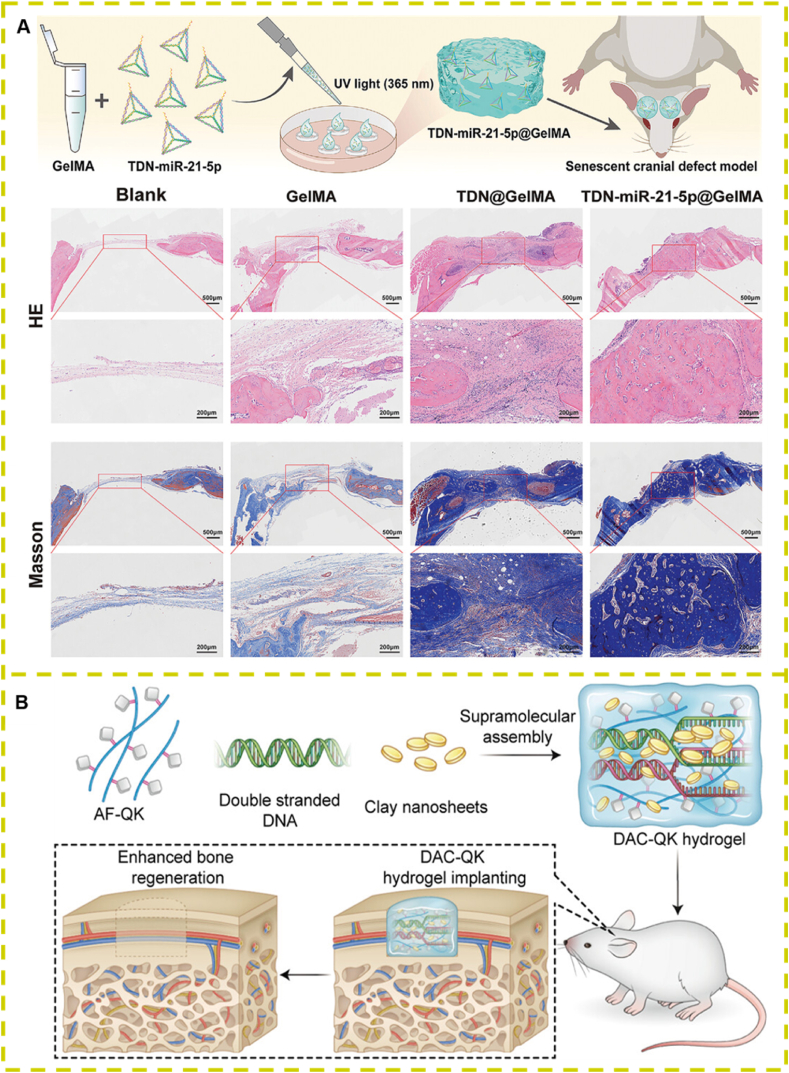
DNA hydrogels for bone regeneration and repair. (A) TDN-miR-21-5p@GelMA to promote vascularized bone regeneration in senescent defects [150]. Copyright 2024, Wiley. (B) Preparation of dual nanoengineered self-Assembled DAC hydrogel for vascularized bone regeneration [151]. Copyright 2025, Springer Nature.

In the context of pathological bone defect repair, corticosteroid-induced upregulation of Dickkopf-1 (DKK1) suppresses Wnt signaling and leads to steroid-associated osteonecrosis (SAON). An injectable lithium-heparin hydrogel has been developed for the co-delivery of miR-335-5p-functionalized tetrahedral DNA nanostructures (MiR@TDNs) and lithium ions. Through targeted inhibition of DKK1 by MiR@TDNs in concert with lithium-mediated activation of the Wnt pathway, this platform effectively promotes osteogenic differentiation of bone marrow mesenchymal stem cells, angiogenesis, and reconstruction of the necrotic microenvironment in SAON models, resulting in substantial bone defect repair. Quantitative analysis results of micro-CT indicated that there was no significant difference in bone volume/total volume (BV/TV) and bone mineral density (BMD) between the Hep-gel and blank control groups, both of which were lower than those in the Li-hep-gel group. This study represents the first integration of miRNA-functionalized TDNs with ionic delivery, offering an innovative synergistic strategy that bridges gene therapy and tissue engineering for complex bone defects [146]. These tFNA hydrogels enable quantitative integration and spatial organization of peptides, nucleic acids, and small-molecule therapeutics. They provide an ideal platform for constructing multifunctional bone repair systems that combine targeted recruitment, intelligent responsiveness, and sequential release. Their intrinsic biocompatibility, tunable degradability, and phosphate backbone-mediated mineralization have been validated across multiple models. For example, tFNA hydrogels functionalized with Apt02 undergo gelation under mild conditions at 37°C, thereby avoiding cell damage associated with conventional hydrogel polymerization at elevated temperature (∼46°C), and significantly promote regeneration in rat critical-sized calvarial defects [147]. Naringin-loaded tFNA-chitosan composite hydrogels markedly enhance alveolar bone defect repair via activation of the BMP/RUNX2/OSX signaling axis [148]. Moreover, TDN coatings on titanium substrates substantially improve cell adhesion, proliferation, osteogenic differentiation, and osseointegration [149], while miR-21-5p-loaded TDNs embedded in GelMA hydrogels overcome impaired osteogenesis-angiogenesis coupling in aged bone defects. 12 weeks post-implantation, micro-CT reconstruction images visually presented superior bone regeneration in TDN-miR-21-5p@GelMA group than other groups with significant higher BV/TV (Fig. 9A) [150]. Despite these promising outcomes, the complex synthesis and functionalization of tFNA hydrogels result in high production costs and batch-to-batch variability, and precise matching of degradation kinetics with the temporal requirements of bone regeneration remains some challenges. Future developments should incorporate dynamic covalent bonds or enzyme-sensitive motifs to enable intelligent degradation control, alongside streamlined synthesis protocols to facilitate industrial-scale translation.

Dynamic DNA hydrogels, characterized by reversible crosslinking networks and stimulus-responsive behavior, offer the capacity to sense the changes in the bone repair microenvironment, thereby aligning material remodeling with cellular proliferation, differentiation, and ECM reconstruction. Their self-healing capability and injectability enable in situ filling of irregular bone defects while maintaining long-term structural integrity. For instance, a dual nanoengineered dynamic DNA hydrogel composed of amyloid fibrils and clay nanosheets has been engineered to mimic the fibrous architecture of native ECM through supramolecular co-assembly, synergistically enhancing mechanical strength. This system exhibits injectability, self-repair, and 3D printability (Fig. 9B) [151]. Similarly, a dynamic GelMA/DNA double-network hydrogel (CGDE) formed via covalent and hydrogen-bond crosslinking is created for biomimetic microenvironment with appropriate mechanical strength and viscoelasticity, facilitating the self-organization of bone marrow mesenchymal stem cells into woven bone-like organoids [152]. Nevertheless, dynamic DNA hydrogels continue to face critical limitations, including insufficient mechanical robustness, mismatched degradation rates relative to bone healing timelines, high manufacturing costs, and unresolved long-term safety concerns, such as genomic integration risks, degradation product toxicity, and immunogenicity. Besides, regulatory approval will require rigorous validation in large-animal models to assess long-term mechanical stability, immune responses, and overall safety.

To further clarify the characteristics, applicable conditions and inherent trade-offs of mainstream DNA hydrogel platforms for bone repair, we summarizes the crosslinking strategies and comprehensive performances of pure DNA hydrogels, DNA-chitosan, DNA-silk fibroin and multi-component composite DNA hydrogels [153,154]. Pure DNA hydrogels possess outstanding biocompatibility and intrinsic stimulus responsiveness, but their insufficient mechanical strength fundamentally restricts their application in load-bearing bone repair. Introducing natural or synthetic polymer networks has become the mainstream optimization strategy. However, such modifications inevitably bring notable trade-offs. For natural polymer composites, such as DNA-chitosan and DNA-silk fibroin, the added polymer network effectively increases crosslink density and mechanical robustness, but the dense intermolecular interactions will restrict the reversible conformational changes of DNA strands, leading to a significant decline in stimulus responsiveness. Excessively high polymer content will also slightly reduce the superior biocompatibility of pure DNA systems. For synthetic polymer hybrid systems, they can achieve the highest mechanical modulus to meet the requirements of heavy load-bearing bone tissues, yet residual crosslinking agents and synthetic matrices may compromise the inherent biosafety of DNA hydrogels. These compromises mean that material design for bone repair needs to strike a balanced trade-off among mechanical performance, biocompatibility and dynamic responsiveness according to the actual load conditions of bone defects.

Autologous bone grafting remains the clinical gold standard for the treatment of critical-sized bone defects owing to its unique combination of osteogenic cells, osteoinductive factors, and osteoconductive matrix. Compared with autografts, DNA hydrogel-based systems offer distinct advantages in terms of injectability, programmability, controlled delivery of bioactive molecules, and reduced donor-site morbidity. Nevertheless, most evidence supporting DNA hydrogel-mediated bone regeneration currently originates from small-animal models, and direct comparisons with autologous bone grafts remain scarce. Therefore, although DNA hydrogels have demonstrated considerable promise as bioactive regenerative platforms, their ability to match or surpass the long-term structural and functional outcomes.

### 5.2 Cartilage regeneration and repair

Relying on the 3D cell encapsulation and microenvironment regulation capabilities of DNA hydrogels, multiple functionalized systems have been developed to address the limited self-repair capacity of cartilage tissue. Cartilage defects exhibit extremely limited intrinsic repair capacity due to the absence of vasculature, innervation, and lymphatic networks, rendering conventional therapeutic strategies largely inadequate. Although optimization of mechanical properties, scalable manufacturing, and long-term safety evaluation remain key barriers to clinical translation, DNA hydrogels continue to demonstrate considerable promise for personalized cartilage repair. The construction of cartilage organoids has emerged as a powerful strategy for cartilage repair. DNA-silk fibroin (DNA-SF) hybrid hydrogels incorporating covalently tethered chondrogenic inducers form a sustained-release system (DSRGT) that maintains optimal chondrogenic phenotypes of cartilage organoids (COs) over a four-week period. With a high porosity of 55-62% and pronounced shear-thinning behavior, these hydrogels support 3D bioprinting and ultraviolet crosslinking-mediated structural stabilization [155]. Microfluidic fabrication of RGD-peptide-modified silk fibroin-DNA double-network hydrogel microspheres (RSD-MS) yield uniformly porous architectures that significantly enhance cell adhesion, proliferation, and chondrogenic differentiation via integrin-mediated signaling. The resulting cartilage organoid precursors (COPs) demonstrate outstanding regenerative efficacy in rat cartilage defect models [156].

**Fig. 10:**
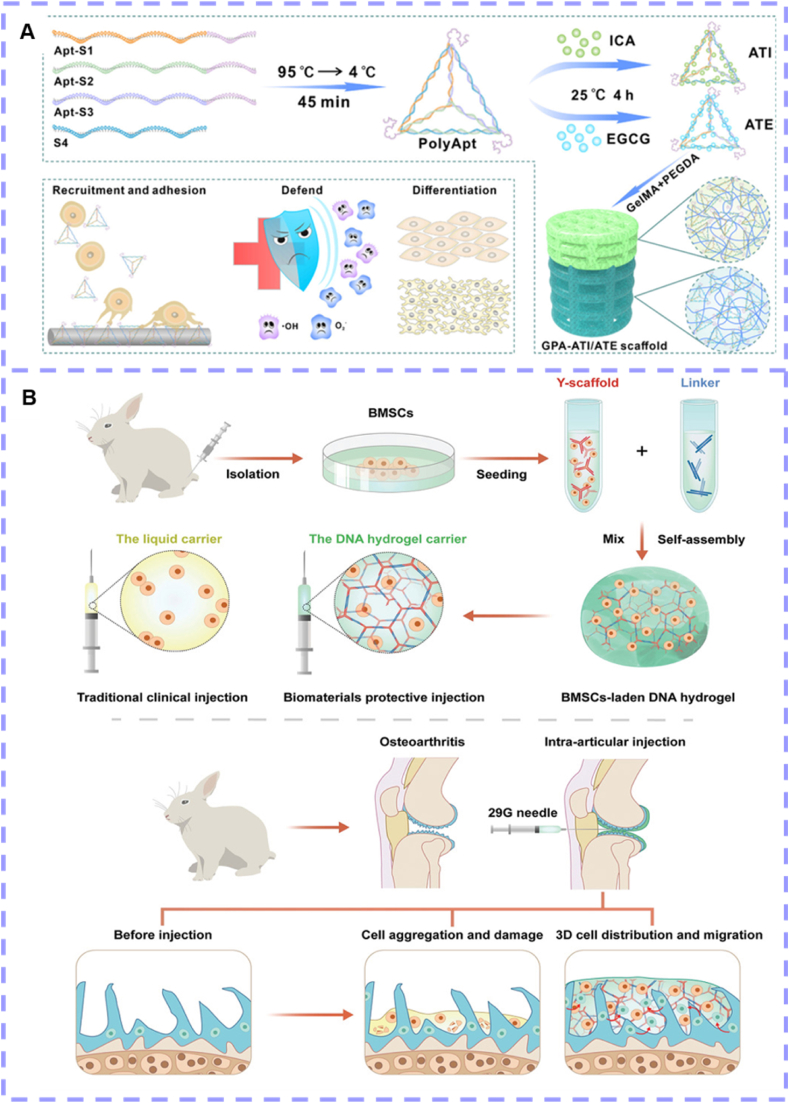
DNA hydrogels for cartilage regeneration and repair. (A) ATI or ATE-functionalized GPA gradient scaffolds for repairing osteochondral defects and promoting chondrogenesis and osteogenic differentiation [157]. Copyright 2025, Wiley. (B) Preparation of DNA supramolecular hydrogels containing BMSCs for improving therapeutic efficacy in severe OA [158]. Copyright 2021, Wiley.

In situ cartilage regeneration strategies further exploit multivalent DNA aptamers to efficiently recruit endogenous stem cells, synergistically eliminate reactive oxygen species via antioxidant components, and guide spatially oriented differentiation using degradable gradient hydrogels. A biomimetic bilayer hydrogel scaffold functionalized with tetrahedral DNA nanoframe aptamers, featuring an upper cartilage-specific layer (ATI) and a lower bone-specific layer (ATE), can enhance stem cell adhesion and migration while mitigating oxidative stress-induced damage. This system provides early mechanical support and concurrently promotes differentiation toward chondrocytic and osteogenic lineages (Fig. 10A) [157]. Despite these advances, such complex architectures are constrained by high synthesis costs, limited scalability, and poorly defined relationships between degradation kinetics and regenerative timelines, thereby restricting clinical feasibility. Future efforts should focus on developing modular plug-and-play DNA nanoplatforms to reduce cost, optimizing smart degradable crosslinking strategies, and validating long-term stability and immunogenicity.

In the context of osteoarthritis (OA), DNA hydrogels exhibit unique advantages by integrating targeted inflammatory modulation with mechanical signaling cues. DNA supramolecular hydrogels used as mesenchymal stem cell delivery systems protect cells from high shear forces within the joint environment, thereby enhancing chondrogenesis and activating regenerative signaling pathways, which significantly improves therapeutic efficacy in severe OA models (Fig. 10B) [158]. Double-network DNA-silk fibroin (SF) hydrogels are developed with precisely tunable stiffness, demonstrating that intermediate mechanics most effectively promotes stem cell chondrogenic differentiation [159]. To address the vicious cycle of persistent inflammation and impaired regeneration in OA, they subsequently engineer a DNAzyme-based intelligent hydrogel system. Constructed via rolling circle amplification to form a self-supporting network, this platform co-delivers inducible nitric oxide synthase (iNOS)-targeting DNAzymes and bone marrow mesenchymal stem cell-derived exosomes, enabling inflammation-responsive release and immune microenvironment remodeling while simultaneously promoting cartilage regeneration [160].

Similar to bone repair scenarios, multiple DNA composite hydrogel systems have been developed for cartilage regeneration [161]. Cartilage tissues require moderate mechanical strength, excellent viscoelasticity and sustained dynamic responsiveness to adapt to joint microenvironments. Pure DNA hydrogels show ideal biocompatibility and stimulus sensitivity but lack enough mechanical support for cyclic compression and friction in joint cavities. DNA-silk fibroin and DNA-chitosan composites are the most widely used candidates for cartilage repair. Compared with pure DNA hydrogels, their reinforced network structure improves mechanical strength and anti-friction capacity, which fits the physiological characteristics of cartilage [162]. Nevertheless, the dense polymer network inevitably limits the dynamic sol-gel transition and target-responsive behavior of DNA sequences. For cartilage repair and osteoarthritis treatment that rely on inflammation-responsive drug release, the loss of partial stimulus responsiveness becomes a prominent drawback. In addition, multi-component hybrid hydrogels with synthetic polymers can further enhance mechanical durability for long-term joint movement, but they also introduce potential biosafety risks and weaken the natural biointerface compatibility of DNA materials [163].

Nevertheless, the manufacturing quality control of such multicomponent systems remains challenging, and concerns persist regarding long-term mechanical stability and intra-articular retention. Future studies should prioritize design simplification through integrated DNA nanostructures, incorporation of cartilage-binding motifs or enhanced crosslinking density to improve anchorage and resistance to degradation, and alignment of material persistence with the chronic progression of OA. DNA hydrogels for cartilage regeneration research have evolved into a multilayered framework encompassing organoid construction, in situ induction, and OA therapy. Continued advancement of modular design strategies and dynamically tunable properties is expected to enable transformative progress in personalized cartilage repair and cell-free regenerative therapies.

### 5.3 Cardiovascular regeneration and repair

The injectability, sustained delivery and self-healing features of DNA hydrogel platforms make them suitable for myocardial infarction and vascular tissue repair [[164], [165], [166]]. Injectable DNA hydrogels can attenuate post-MI ventricular dilation by reducing ventricular wall stress, while also serving as carriers for encapsulated stem cells, whose retention and survival rates are markedly improved compared with direct cell injection.

**Fig. 11:**
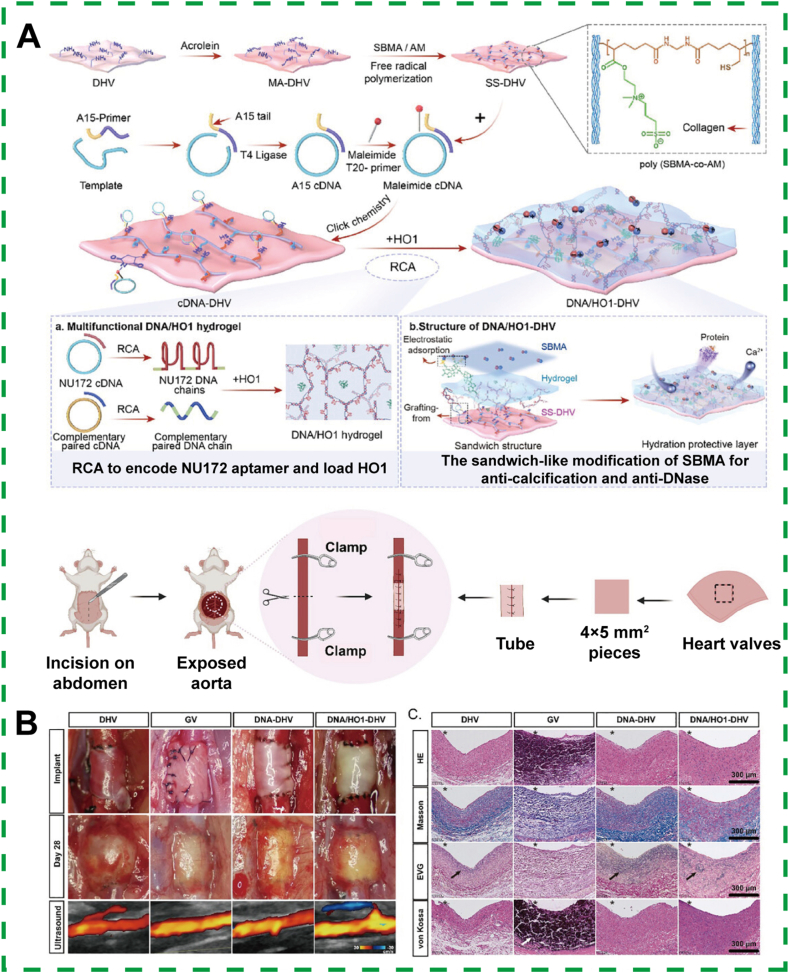
DNA hydrogel in cardiovascular regeneration and repair. (A) Preparation process for DHV (DNA/HO1-DHV) in immunomodulator factory retrofitting and schematic diagram of the ventral and aortic transplantation model. (B) Doppler ultrasound examination analysis and staining results of intracorporeal cardiac valve regeneration [53]. Copyright 2025, Wiley.

The sequence specificity of DNA hydrogels further enables growth factor delivery with precisely programmable release kinetics, thereby promoting neovascularization and myocardial protection. For example, grafting DNA hydrogels onto decellularized heart valves (DHVs) via customized rolling circle amplification (RCA) has shown to facilitate heart valve regeneration (Fig. 11) [53]. In vascular tissue engineering, physically stimulus-responsive DNA hydrogels demonstrate robust angiogenic potential, inducing capillary-like network formation by human umbilical vein endothelial cells (HUVECs) in vitro and significantly enhancing vascular regeneration *in vivo*. These effects stem from the efficient gene and drug delivery capacity of DNA hydrogels, which enables sustained, localized release and thereby amplifies tissue repair efficacy. Nevertheless, clinical translation will require resolution of challenges related to scalable manufacturing, long-term degradation behavior, and comprehensive *in vivo* immunogenicity assessment. Collectively, these studies highlight a paradigm shift in DNA hydrogels from passive carriers to active regulators of the immune microenvironment, establishing a broadly applicable platform for next-generation biomaterials that integrate therapeutic activity with regenerative induction. Conventional 3D-printed scaffolds often fail to recapitulate the dynamic bioactive microenvironment of native ECM, limiting their ability to regulate cell behavior and promote functional tissue regeneration. To address this limitation, dynamic DNA hydrogels reinforced with black phosphorus nanosheets (BPNSs) have been integrated with 3D-printed scaffolds to create gel-scaffold composites that combine mechanical support with biological functionality [167]. The DNA hydrogel confers self-healing capability and reversible network dynamics, while noncovalent interactions between BPNSs and bioactive factors enable sustained release of angiogenic growth factors (VEGF). This coordinated delivery modulates endothelial angiogenesis and mesenchymal stem cell differentiation, effectively overcoming the bioinert nature and static microenvironment of conventional 3D-printed scaffolds. By leveraging the programmability of DNA hydrogels and the functional integration of nanomaterials, this strategy provides an extensible platform for regenerative medicine targeting cardiovascular and other vascularized tissues. Nonetheless, key translational barrier, including scalable fabrication, long-term *in vivo* degradation kinetics, and clinical-grade standardization, remain unresolved. Future advances will likely require integration with synthetic biology and precision medicine to develop personalized, injectable DNA hydrogel systems capable of addressing more complex cardiovascular pathologies, such as atherosclerosis and MI.

The core value of DNA hydrogels in cardiovascular regeneration can be summarized in three principal dimensions: (i) Provision of ECM-like 3D scaffolds that support cell adhesion and proliferation; (ii) restoration of myocardial electrical activity through conductive or stimulus-responsive mechanisms; and (iii) programmable delivery of bioactive factors to enhance angiogenesis and anti-inflammatory immunomodulation. Together, these attributes position DNA hydrogels as versatile and customizable platforms offering multilayered regenerative solutions for heart valve repair, myocardial defects, and vascular regeneration.

### 5.4 Skin regeneration and repair

**Fig. 12:**
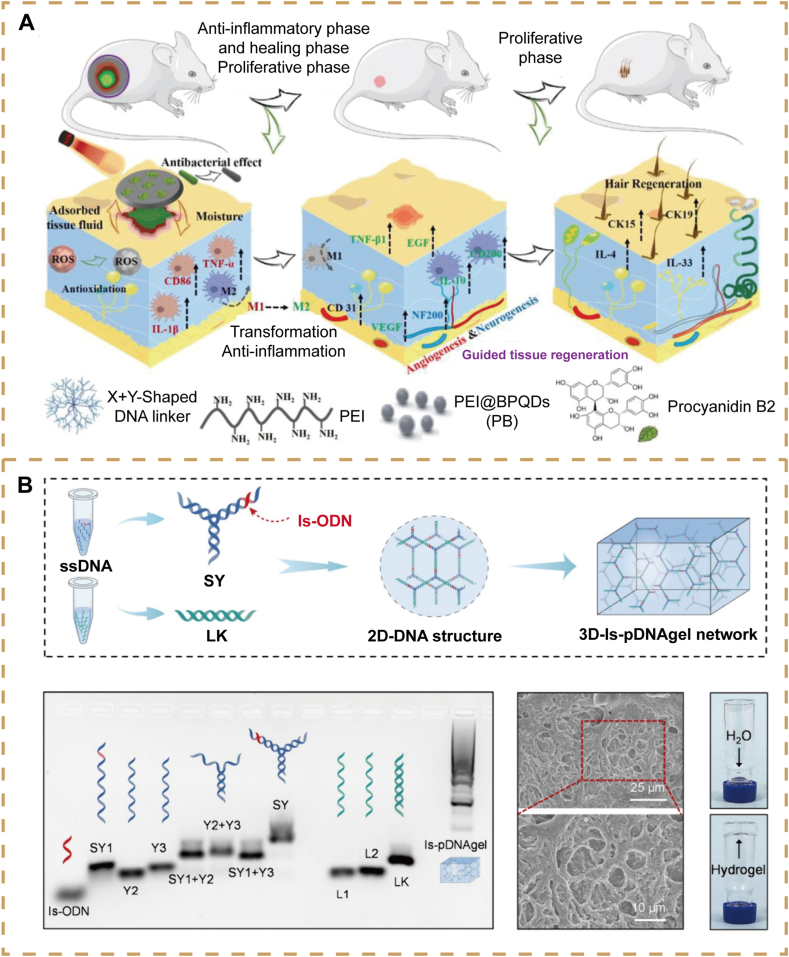
DNA hydrogel for skin regeneration and repair. (A) Is-pDNA gel construction and related gel electrophoresis analysis, SEM imaging, and digital images [171]. Copyright 2025, American Chemical Society. (B) Schematic illustration of OPC B2/PBCD gel promoting the healing of diabetic infectious wounds [35]. Copyright 2021, Wiley.

Although clinical investigations of DNA hydrogels for skin repair remain limited, their intrinsic physicochemical properties closely align with the fundamental requirements of wound healin [168]. Diabetic infected wounds represent one of the most challenging categories of chronic wounds, necessitating dressings with high absorptivity, customizable form factors, rapid self-healing, and multifunctionality. Owing to their immunomodulatory, pro-angiogenic, and antibacterial capabilities, DNA hydrogels can effectively reverse chronic inflammatory microenvironments and accelerate skin tissue regeneration [169,170]. For instance, multifunctional dressings via dynamic crosslinking between DNA motifs and polyethyleneimine, loaded with BP QDs and proanthocyanidin B2, have been shown to induce macrophage M1-M2 polarization, activate neurovascular regeneration, and recruit bone marrow-derived cells to stimulate adaptive immune responses, thereby markedly promoting tissue and hair follicle regeneration in diabetic infected wounds (Fig. 12A) [171]. To improve clinical convenience, a sprayable TR21@TS nucleic acid hydrogel integrating hemostatic, immunomodulatory, and regenerative functions has been developed, which effectively alleviates oxidative stress and inflammation, accelerates wound closure, and reduces scar formation [172]. To address persistent overexpression of inflammatory mediators, an immunosuppressive pure DNA hydrogel (Is-pDNAgel) is designed to sequester chemokines via high-density negative charges while simultaneously blocking pro-inflammatory signaling pathways through immunosuppressive domains. This dual mechanisms significantly enhance angiogenesis and accelerate chronic wound healing, outperforming commercially available dressings (Fig. 12B) [35]. Furthermore, the first triboelectric-responsive DNA hydrogel combined with conductive polymers enables pulsed electrical stimulation to remodel bioelectrical signaling and activate cellular electrotaxis, achieving a 99.8% wound closure rate within 14 days in bacterially infected diabetic wounds, accompanied by enhanced collagen deposition and hair follicle regeneration [173]. With advances in 3D printing and AI, functional inks incorporating salmon sperm DNA and DNA-induced biosilica have been developed, enabling machine learning-guided customization of personalized dressings. These systems synergistically eliminate reactive oxygen species (ROS), promote angiogenesis, and suppress inflammation, thereby significantly accelerating healing of both acute and diabetic wounds [70].

Pioneering studies employing tFNAs as self-assembled DNA nanomaterials demonstrate that activation of AKT signaling pathway markedly enhances keratinocyte and fibroblast proliferation and migration, providing the therapeutic potential of nucleic acid nanomaterials for scar-free skin regeneration [[174], [175], [176]]. These editable DNA nanostructures thus represent a safe and efficient strategy for skin tissue regeneration [177]. In the context of burn wounds, which are characterized by persistent ROS-driven inflammation and insufficient mechanical support, a tetrahedral framework nucleic acid-based DNA hydrogel is developed by diamond lattice architectures. This system scavenges ROS and modulates the NF-κB pathway, thereby reshaping the immune microenvironment and accelerating burn wound healing [31]. Nevertheless, tFNA-based hydrogels continue to face limitations, including insufficient mechanical strength, poor long-term *in vivo* stability, and potential immunogenicity. Future efforts should focus on enhancing mechanical performance through topological optimization and crosslink density regulation, while integrating functional modules such as miRNAs and peptides to achieve synergistic multimodal therapy. Overall, DNA hydrogels for skin repair have progressed from proof-of-concept demonstrations toward intelligent and personalized therapies targeting complex wounds, such as diabetic ulcers and burns.

### 5.5 Nerve regeneration and repair

Combined with the stimulus-responsive and immunomodulatory delivery functions of DNA hydrogels, a series of dressings have been developed for acute wounds, diabetic infected wounds and burn repair. Multifunctional DNA hydrogels have further been demonstrated to simultaneously modulate immune responses and activate neuronal repair programs, thereby accelerating neural regeneration while promoting tissue repair. Such dual-mode regenerative capacity is particularly relevant for complex tissue defects, including diabetic infected wounds. For example, a hydrocolloid-cotton structured DNA hydrogel is fabricated via dynamic crosslinking between grafted DNA motifs and polyethyleneimine with BPQDs, which exhibits both nerve tissue repair and regeneration functions [171]. Owing to their programmable degradation and excellent biocompatibility, DNA materials are well suited for temporally controlled neurotrophic delivery. An XT-type DNA hydrogel loaded with VEGF and NGF exploits the differential degradation kinetics of X- and T-shaped DNA motifs to achieve biphasic release profile [178]. This system significantly enhances cell proliferation, migration, and myelination while maintaining high cell viability, and further demonstrates superior repair of long-gap peripheral nerve injuries compared with conventional chitosan. Nevertheless, although its regenerative outcomes in animal models surpass those of traditional conduits, electrophysiological recovery and myelinated nerve fiber density remain inferior to autologous nerve grafts, which continue to represent the clinical gold standard for bridging large peripheral nerve defects. The superiority of autologous nerve grafts is primarily attributed to their preserved extracellular matrix architecture, native Schwann cell population, and intrinsic neurotrophic microenvironment. Consequently, future development of DNA hydrogel systems should focus not only on enhancing axonal regeneration but also on recapitulating these biological features to achieve clinically competitive outcomes. Additional translational challenges include relatively high production costs and the need for long-term safety validation. Despite these limitations, the simplicity of fabrication and biomimetic release behavior underscore its considerable potential for further optimization.

**Fig. 13:**
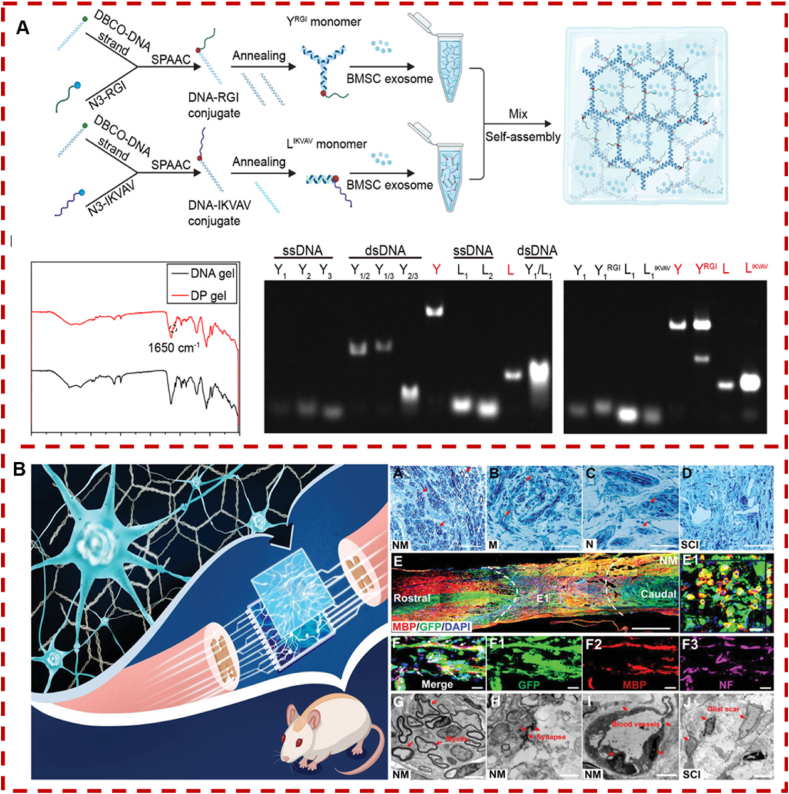
DNA hydrogel for nerve regeneration and repair. (A) Synthesis of programmable DNA-peptide conjugated exosome-loaded (DPE) hydrogels, FTIR spectra and PAGE characterization of DNA hydrogels and DNA-peptide (DP) hydrogels [179]. Copyright 2025, Wiley. (B) Regenerative neural network formed by DNA supramolecular hydrogels supported at the lesion site and the staining of myelin regeneration [180]. Copyright 2021, Wiley.

Existing DNA-based therapeutic platforms often suffer from limited capacity to stably integrate multiple bioactive cues, thereby constraining precise regulation of Schwann cell (SC) repair phenotypes required for sustained regenerative function. To address this limitation, a programmable DNA-peptide conjugated hydrogel constructed via click chemistry is developed to precisely integrate neurotrophic-mimetic peptides, laminin-derived peptides, and bone marrow mesenchymal stem cell-derived exosomes, enabling sequential release and fine-tuned control of multiple therapeutic components (Fig. 13A) [179]. This hydrogel can reprogram SCs toward a pro-regenerative phenotype through activation of the Nrg1/ErbB signaling pathway, thereby promoting axonal regrowth and angiogenesis. In a rat sciatic nerve injury model, the system undergoes in situ gelation and significantly accelerates functional recovery, offering an efficient and clinically relevant strategy for peripheral nerve regeneration. Beyond peripheral nerves, highly permeable DNA supramolecular hydrogels showed to promote neurogenesis and functional recovery following complete spinal cord transection (Fig. 13B) [180]. Combining self-healing capability with appropriate mechanical support, these materials satisfy key criteria for ideal implantable scaffolds and exhibit strong translational promise. Moreover, the injectability of DNA hydrogels enables minimally invasive delivery to fill irregular lesion cavities and provide a permissive substrate for axonal extension. Nevertheless, the clinical translation of DNA hydrogel-mediated neural regeneration remains contingent upon validation in large-animal models, including rigorous functional outcome assessment, demonstration of scar-free biodegradation without glial scar formation, and integration with electrical stimulation paradigms to further enhance neural repair. In addition, the pivotal preclinical regenerative studies covering animal models, defect types, DNA hydrogel compositions, and primary efficacy endpoints in various tissues are summarized in Table 4.

**Table 4:** The pivotal preclinical regenerative studies covering animal models, defect types, DNA hydrogel compositions, and primary efficacy endpoints.

Tissue	Animal model	Defect type	DNA hydrogel composition	Primary outcome	Reference
Bone	Rat	Calvarial defect	Enzyme-responsive DNA-PEG hydrogel	The hydrogel significantly enhances vascular network formation, osteogenic marker expression, and mineral deposition relative to controls	145
Bone	Rabbit	Femoral head defect	An injectable heparin lithium hydrogel with MiR335-5p-pendant tetrahedron DNA nanostructures	The hydrogel regulates the Wnt pathway, resulting in the promotion of osteogenic differentiation and improvement of angiogenesis, enhancing bone regeneration and microcirculation reconstruction in osteonecrosis.	146
Bone	Rat	Calvarial defect	TDN-miR-21-5p@GelMA scaffold	TDN-miR-21-5p could promote osteogenic differentiation and alleviate senescent-related phenotype of O-BMSCs, simultaneously improving angiogenic capacity of O-EPCs, TDN-miR-21-5p@GelMA scaffolds are effective in repairing senescent critical-size bone defects with significantly increased osteogenic- and angiogenic-related markers expression	150
Cartilage	Rat	Osteoarthritis (OA)	D bioprinting of a DNA-silk fibroin (DNA-SF) hydrogel sustained-release system (DSRGT) with bone-marrow mesenchymal stem cells (BMSCs)	The hydrogel promotes the chondrogenic differentiation of BMSCs and inhibits their fibrotic and hypertrophic progression during long-term culture	155
Cartilage	Sprague-Dawley rats	Osteoarthritis (OA)	A DNAzyme-based smart hydrogel delivering iNOS-targeting DNAzymes and BMSC-derived exosomes	The hydrogel could effectively reduce pro-inflammatory NO production, promote M2 macrophage polarization, and enhance chondrocyte proliferation and extracellular matrix restoration	160
Myocardium	Rat	A rat abdominal aorta implantation model	A grafting-from DNA hydrogel that encodes thrombin-binding aptamers (NU172) for trapping heme from blood while encapsulating heme oxygenase-1 (HO-1)	This hydrogel is constructed on decellularized heart valves (DHVs) via rolling circle amplification (RCA), BV functions as a scavenger of ROS and an anti-inflammatory agent, promoting M2-type macrophage polarization, inducing phosphorylation of endothelial nitric oxide synthase (eNOS), releasing nitric oxide (NO), and activating the PI3K-AKT signaling pathway to enhance valve endothelialization	53
Skin	Mouse	Diabetic infectious wounds	Biologically active DNA Hydrogels loading with BPQDs-doped cationic polymer polyethyleneimine (PEI) and wound healing nutrient procyanidin B2	The hydrogel promotes skin tissue healing, guide macrophage polarization, skin nerve regeneration, and tissue regeneration	171
Peripheral nerve	Rat	Sciatic nerve injury	A programmable DNA-peptide conjugated, exosome-loaded (DPE) hydrogel	The DPE hydrogel activates the Nrg1/ErbB/PI3K/Akt pathway and promotes their reprogramming into a repair phenotype, as well as enhances their proliferation and migration	179

### 5.6 Cancer therapy and cancer-resected tissue regeneration

**Fig. 14:**
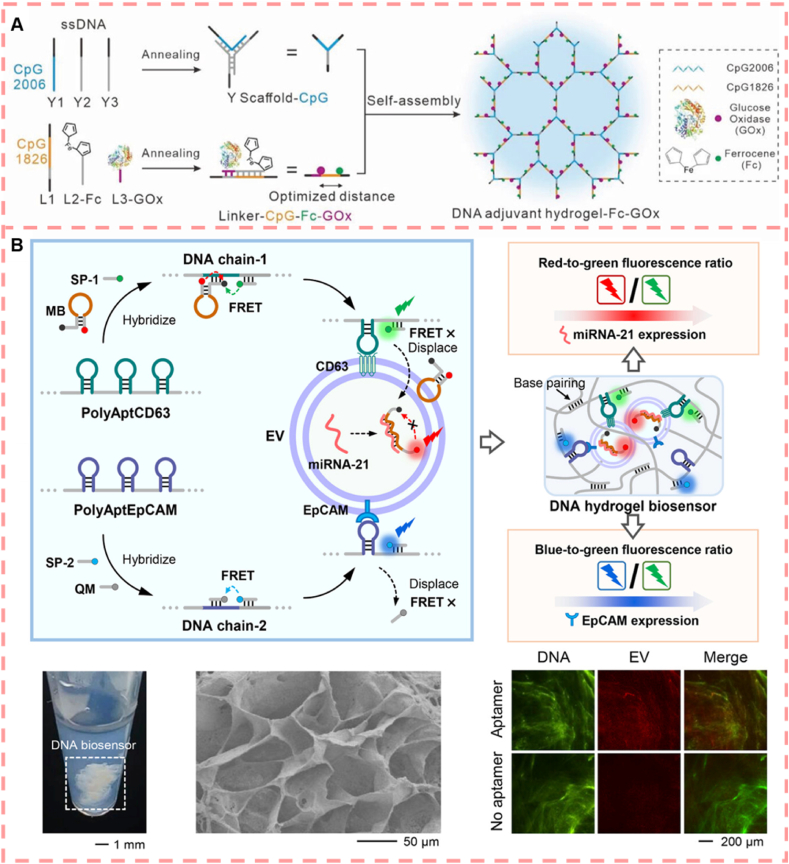
DNA hydrogel for cancer therapy and cancer-resected tissue regeneration. (A) DNA adjuvant hydrogel scaffold enzyme cascade reaction for tumor CDT and immunotherapy [184]. Copyright 2024, Wiley. (B) Engineering supramolecular hydrogel biosensors using DNA nanotechnology for detecting miRNA-21 and EpCAM in extracellular vesicles. Images of the synthesized hydrogel biosensor in a tube, SEM images of the biosensor and fluorescence images [185]. Copyright 2025, Wiley.

Integrated with biosensing, targeted delivery and stimulus-responsive modules, DNA hydrogels realize the combination of tumor treatment and postoperative tissue reconstruction. For resectable solid tumors, injectable DNA hydrogels can be implanted directly into the post-operative cavity to eradicate residual malignant cells while simultaneously serving as a regenerative scaffold, thereby enabling a seamless integration of tumor ablation and tissue repair [181]. Some studies have demonstrated that immunotherapy can serve as one of the effective approaches for tumor treatment [182,183]. A spatially confined catalytic architecture enhances reaction efficiency by approximately nine-fold compared with the corresponding free systems, markedly amplifying chemodynamic therapy (CDT)-induced immunogenic cell death (Fig. 14A) [184]. In parallel, DNA hydrogels equipped with targeting aptamers enable effectively reduce off-target toxicity. A rational design of DNA aptamer hydrogels (Apt-GelMA) with gelatin polymer chains modified with anti-HGF (hepatocyte growth factor) c-Met aptamer and anti-EGFR (epidermal growth factor receptor) CL-4 aptamer, as effective post-surgery dressing materials, enhancing their clinical availability. Our Apt-GelMA hydrogel dressing offers a promising solution for tumor post-surgery rehabilitation by enhancing both tumor suppression and tissue repairing [186].

DNA supramolecular hydrogels are 3D networks formed through noncovalent self-assembly of DNA motifs, integrating the molecular recognition precision and biocompatibility of nucleic acids with the softness and porosity characteristic of hydrogels. The dynamic reversibility of supramolecular interactions further endows these systems with tumor microenvironment–responsive drug release capabilities, including sensitivity to pH and enzymatic cues [187]. A major technical bottleneck lies in the aggregation-induced inactivation of DNA/PEI polyplexes when loaded into hydrogels. To address this limitation, a cage-nanoparticle encapsulation (CnE) strategy enables aggregation-free packaging of highly concentrated nonviral vectors (up to 5 μg/μL), while preserving potent transfection activity across diverse hydrogel matrices, including PEG, HA, and fibrin, thereby substantially broadening the applicability of hydrogel-based gene therapy [188]. Nonetheless, CnE systems still face challenges, such as restricted diffusion and HA-induced aggregation/dissociation-driven heterogeneity in *in vivo* transfection. Future efforts should focus on improving encapsulation uniformity, developing material-specific delivery strategies, and implementing active release mechanisms to balance local retention with efficient tissue penetration. Injectable DNA supramolecular hydrogel vaccines (DSHV) have been designed to recapitulate lymph-node-like microenvironments, leveraging high local concentrations of CpG motifs to recruit and activate antigen-presenting cells and to elicit robust TH1/TH2-biased antitumor immune responses [189]. A defining advantage of this platform is its sequence programmability, which allows the co-loading of chemotherapeutic agents, therapeutic genes, and immunomodulators, thus enabling a shift from localized tumor control toward systemic immune modulation.

In the diagnostic arena, DNA-based biosensors have been developed to integrate extracellular vesicle recognition, enrichment, and dual-marker detection of miRNA-21 and EpCAM, achieving 100% diagnostic accuracy for breast cancer within 30 min. Their 3D porous architecture, combined with multivalent aptamer presentation, significantly enhances capture efficiency, while cascade assembly mechanisms amplify detection signals. Moreover, freeze-drying fabrication simplifies clinical operation and deployment (Fig. 14B) [185]. Further integration of hybridization chain reaction-based signal amplification, optimized aptamer engineering, and portable fluorescence readout devices is expected to enhance clinical feasibility. Although DNA hydrogels exhibit distinctive advantages in tumor therapy and tumor-resected tissue regeneration, their clinical translation remains challenges related to carrier uniformity, controllable *in vivo* distribution, and long-term biosafety. Future advances integrating precision drug delivery, cell-based therapies, and immune modulation are anticipated to drive tumor diagnosis and treatment toward increasingly personalized, minimally invasive, and highly efficient clinical paradigms.

## 6 Practical challenges and clinical translation analysis of DNA hydrogels

Despite the remarkable progress achieved in DNA hydrogels, several fundamental limitations continue to hinder their clinical translation [190,191]. First, the immunogenicity of DNA-based materials remains incompletely understood [192]. While CpG-containing motifs are intentionally employed to activate TLR9 signaling in vaccine delivery and immunotherapy, unintended activation of innate immune pathways may become problematic in non-TLR-targeted applications such as tissue regeneration, drug delivery, and long-term implantation [193]. The extent to which CpG density, degradation products, and prolonged exposure contribute to chronic inflammation or off-target immune responses remains insufficiently characterized. What's more, challenges related to degradation stability and biosafety are equally prominent. DNA hydrogels are inherently susceptible to nuclease degradation in physiological environments, compromising their functional longevity *in vivo*. Although chemical modifications (e.g., Morpholino) [120] and physical protection strategies (e.g., spermine condensation, poly-L-lysine coating) [77] have been developed to enhance nuclease resistance, the long-term metabolic fate, immunogenicity of modified derivatives, and risk of organ accumulation remain largely unexplored. Current evidence only indicates negligible systemic toxicity or organ damage after short-term local implantation, with no obvious cytotoxicity observed in vitro, but long-term safety data are lacking. Additionally, large-animal studies that support clinical translation are critically insufficient. Data on biodegradation, biodistribution, and chronic immune responses in porcine and ovine models are scarce, representing a major barrier to regulatory approval. Moreover, translational readiness varies considerably across indications: topical wound dressings and localized drug delivery are closest to clinical use due to modest mechanical requirements, limited systemic exposure, and existing regulatory precedents for hydrogel products; whereas load-bearing bone regeneration and spinal cord repair remain the most challenging targets, requiring long-term structural stability, controlled degradation, durable functional recovery, and extensive validation in large animals.

In terms of preclinical and exploratory clinical studies, no pure DNA hydrogels have entered formal Phase I-III clinical trials to date; most work remains at the preclinical stage and the large-scale commercialization has not been achieved. DNA-based composite scaffolds for cranial and long-bone defects have completed large-animal validation [194]. Injectable DNA hydrogels for local drug delivery are still limited to preclinical research, with no registered clinical use. In practical administration, skin wound dressings are applied at a thickness of 1-3 mm and for nerve and cartilage repair, single injection volumes are controlled within 0.1-0.4 mL to avoid excessive tissue compression.

Regarding production and storage, the cost of synthetic DNA (e.g., aptamers, branched DNA) remains high, with laboratory-grade high-purity DNA costing approximately hundreds to thousands of dollars per gram. In contrast, industrial production using natural DNA (e.g., salmon sperm) can reduce the cost. Notably, a recently developed self-template-primer-driven isothermal amplification technique requires only short primers (e.g., 12 nt) at 50 nM concentration and achieves ultra-rapid tandem nucleic acid replication in approximately 30 min at 65°C without additional templates, offering a promising low-cost route for DNA framework hydrogel synthesis [195]. For sterilization, autoclaving, gamma irradiation, and electron beam irradiation break DNA strands, and ultraviolet light is only suitable for surface disinfection of thin materials. Sterilization methods are expected to be developed to maintain the integrity and functionality of hydrogels [196]. For storage, lyophilized hydrogels require refrigeration. Aseptic wet hydrogels are at risks of nuclease degradation and microbial contamination, making room-temperature storage a prominent technical hurdle in the long term. DNA hydrogels are typical combination products, and their classification depends on function: they are defined as medical devices when serving solely as physical scaffolds or dressings, classified as biological products when loaded with nucleic acids, cells, or bioactive components, and regulated as drug-device combination products if integrated with targeted therapeutics. Divergent classification criteria lead to distinct approval pathways and review timelines, further impeding clinical translation.

The future success of DNA hydrogels will depend not only on improvements in responsiveness, programmability, and multifunctionality, but also on addressing core translational barriers including unresolved immunogenicity, insufficient large-animal safety validation, scalable manufacturing, and regulatory clarity. Integrating these requirements with continued innovations in molecular engineering and intelligent material design will be essential for realizing clinically viable DNA hydrogel technologies.

## 7 Conclusions and future perspectives

The progress of DNA hydrogels is systematically summarized from molecular design and synthesis strategies to biological functions and their potential in tissue repair and regeneration. DNA hydrogels, as a class of intelligent biomaterials, integrate high programmability, precise molecular recognition, and excellent biocompatibility of nucleic acids with high water content, biomimetic 3D network structure, and tunable mechanical properties. Through rational design of DNA sequences and crosslinking strategies, the physicochemical properties of DNA hydrogels (mechanical strength, pore size, degradation kinetics, and stimulus responsiveness) can be precisely regulated at the molecular level [197]. In terms of construction methods, pure DNA hydrogels rely on physical interactions or chemical crosslinking, while hybrid DNA hydrogels incorporate polymers, nanomaterials, or bioactive molecules to overcome the limitations of pure DNA systems in mechanical performance and functional expansion, significantly broadening their application scenarios. In tissue engineering, DNA hydrogels not only serve as 3D cell culture scaffolds that support stem cell adhesion, proliferation, and differentiation but also enable precise spatiotemporal control of drug and gene delivery through programmable release mechanisms, achieving targeted modulation of local microenvironments. Their intelligent response capabilities further allow dynamic adaptation to complex pathological microenvironments, providing new strategies for the treatment of inflammation, tumors, and other challenging conditions. With regard to therapeutic translation, DNA hydrogels have shown broad application potential in bone, cartilage, cardiovascular, skin, and neural tissue regeneration, and play the crucial role in cancer therapy and cancer-resected tissue regeneration, demonstrating their capacity to transition from passive structural scaffolds to active multifunctional platforms. However, current DNA hydrogel design largely relies on repeated experimentation and empirical accumulation, whereas AI-assisted DNA sequence design and performance prediction holds the potential to fundamentally enhance efficiency. An integrated design strategy is developed for hydrogels to combine data mining, experimental synthesis, and machine learning from an initial dataset of 180 biomimetic hydrogels by machine learning, thus achieving adhesion strengths exceeding 1 MPa [198]. This intelligent design-prediction-optimization-validation approach is equally applicable to DNA hydrogels by training deep learning models to learn mapping relationships between vast datasets of sequence-structure-performance correlations and predict optimal DNA sequences.

When compared with mainstream natural and synthetic hydrogel counterparts for regenerative medicine, DNA hydrogels exhibit irreplaceable competitive edges. Distinct from collagen, hyaluronic acid, chitosan and other natural hydrogels that lack precise molecular manipulation, and PEG, GelMA, PLA-based synthetic hydrogels with insufficient biological recognition ability and potential cytotoxic risks from crosslinking agents, DNA hydrogels combine excellent biocompatibility, inherent molecular recognition capability, unlimited sequence programmability, and multi-stimulus responsiveness. These unique merits make them ideal platforms for precise tissue engineering, targeted drug delivery and intelligent immunomodulation.

## Ethical statement

As this article is a review paper, we did not need any “ethics approval” and also there was no need for “consent to participate”.

## Funding

This work was supported by 10.13039/501100001809National Natural Science Foundation of China (No. 82302401 to Y.W., and 22304002 to X.Z.), and the 10.13039/100008086Global Joint Research Program funded by the 10.13039/501100002644Pukyong National University (202506290001 to E.B.L.).

## CRediT authorship contribution statement

**Shihui Xu:** Writing – original draft. **Eon-Bee Lee:** Supervision, Validation, Visualization. **Yiming Shen:** Supervision, Validation, Visualization. **Yongtao Wang:** Writing – original draft, Writing – review & editing. **Xue Zhang:** Funding acquisition, Visualization, Writing – original draft, Writing – review & editing.

## Declaration of competing interest

The authors declare that they have no known competing financial interests or personal relationships that could have appeared to influence the work reported in this paper.

## Data Availability

Data will be made available on request.
